# From α-Bromomethylbutenolide to Fused Tri(Tetra) Cyclic Dihydrofurandiones through Barbier Reaction–Heck Arylation Sequence

**DOI:** 10.3390/molecules22122171

**Published:** 2017-12-08

**Authors:** Arbia Talbi, Anne Gaucher, Flavien Bourdreux, Jérôme Marrot, Mohamed L. Efrit, Hédi M’Rabet, Damien Prim

**Affiliations:** 1 Laboratoire de Synthèse Organique Sélective et Hétérocyclique-Evaluation de l’Activité Biologique, Faculté des Sciences de Tunis, Université de Tunis El Manar, Tunis 2092, Tunisie; arbiatalbi1@gmail.com (A.T.); medlotfi.efrit@gmail.com (M.L.E.); 2 Institut Lavoisier de Versailles, Université Paris-Saclay, UVSQ, CNRS, 78035 Versailles, France; anne.gaucher@uvsq.fr (A.G.); flavien.bourdreux@uvsq.fr (F.B.); jerome.marrot@uvsq.fr (J.M.)

**Keywords:** bromomethylbutenolide, tri(tetra)cyclic architectures, Barbier, intramolecular Heck reaction

## Abstract

A Barbier reaction–Heck arylation sequence from α-bromomethylbutenolide to fused tri and tetracyclic lactones has been developed. The first step involving a Barbier reaction enabled installing *ortho*-bromoaromatics in α-ylidene γ-lactones. The latter substrates were subjected to intramolecular Heck reaction conditions which selectively afforded 6,5,5 or 6,6,5 fused ring systems depending on the nature of the base employed.

## 1. Introduction

**Figure 1:**
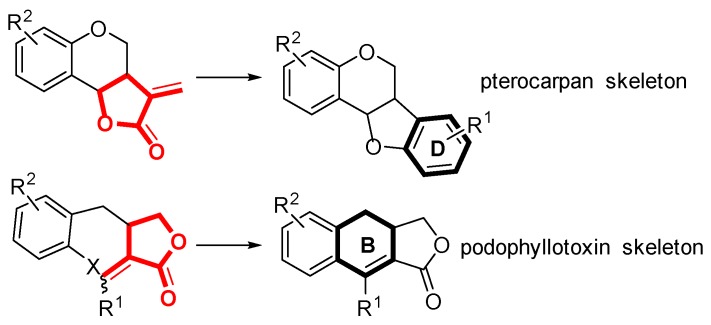
α-Ylidene γ-lactone as advanced intermediates in the synthesis of polycyclic skeletons.

The α-ylidene γ-lactone subunit can be found in a myriad of biologically active compounds [1,2]. The conjugated *exo*-vinylidene fragment is believed to be essential to a wide array of biological activities and thus drove the development of numerous preparation methods [3,4,5,6,7]. The interest of the scientific community for such lactones also stems from their use as intermediates in the synthesis of complex and polycyclic molecular architectures. As examples, the synthesis of the pterocarpan [8,9] and the podophyllotoxin skeletons illustrate α-methylidene butyrolactones as key intermediates for the construction of the final ring D and central ring B, respectively (Figure 1) [10,11,12,13].

**Figure 2:**
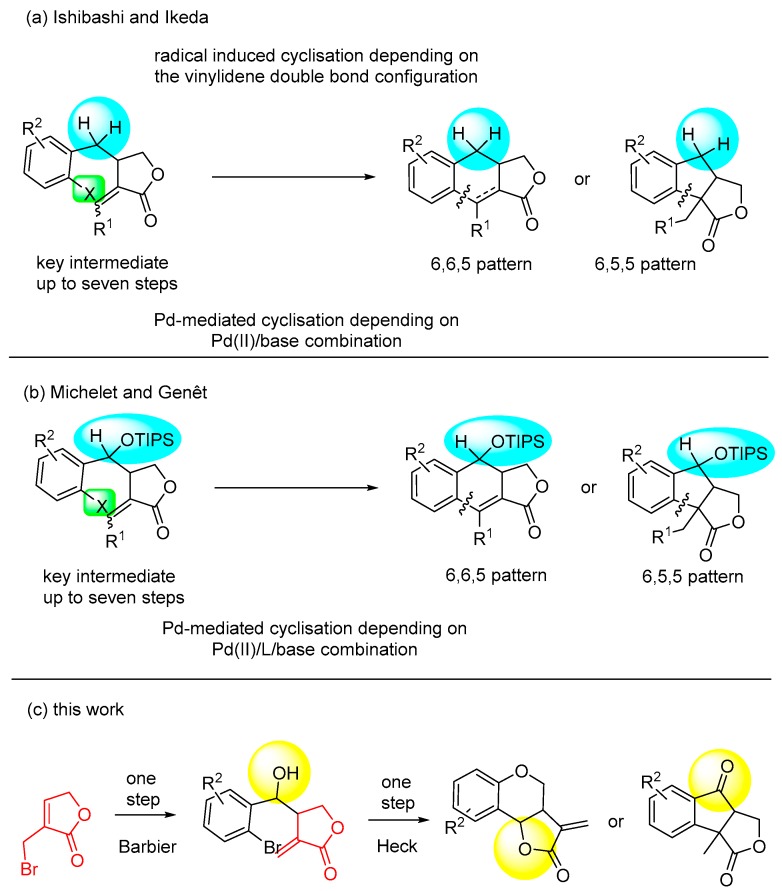
Selective access to 6,6,5 or 6,5,5 polycyclic pattern from α-ylidene γ-lactone. (**a**) Previous work from Ishibashi and Ikeda; (**b**) Previous work from Michelet and Genêt; (**c**) Present work.

Although starting from α-ylidene γ-lactone precursors provided an elegant approach to polycyclic architectures, in each of the latter cases, the preparation of α-methylidene butyrolactone intermediates bearing the mandatory *ortho*-halide substituent (Figure 2a, highlighted in green) required multistep sequences which somewhat hampered the overall access to polycyclic targets [10,11,12,13]. In this context, access to the podophyllotoxin derivatives was carried out mainly on two substrates which are characterized either by the absence of substituents [10,11] or by the presence of a OTIPS group [12] located at the benzylic site which connects the two fragments of the precursor (Figure 2b, highlighted in blue). Two different routes leading to podophyllotoxin derivatives have been studied involving a radical-induced cyclization, depending on the configuration of the vinylidene double bond and a Pd-mediated ring closure depending on the catalytic system used and the substitution at the benzylic site. Indeed, in the presence of a radical cyclisation agent, the Z-isomer led to a mixture of “6,6,5” and “6,5,5” architectures. In contrast, the E-isomer gave an exclusive access to the 5-membered central ring (Figure 2a).

Two different “6,6,5” and “6,5,5” architectures have also been obtained from Pd-catalyzed processes. Without a substituent at the benzylic site, the 6,5,5-pattern was obtained as the sole product from the Z-isomer using K_2_CO_3_ or Et_3_N in combination with Pd(II)/PPh_3_ as the catalytic system. Under these conditions, the E-isomer only led to complex mixtures. In contrast, the use of Pd(II)/PPh_3_, K_2_CO_3_, TlOAc and HCO_2_Na as the hydride source gave the 6,6,5-pattern as the exclusive product (Figure 2a). In the presence of a OTIPS group, the issue of the Pd catalyzed cyclisation process was strongly dependant on the base used (Figure 2b). When Hünig’s base was used, the 6,5,5 pattern was isolated. Mixtures of both patterns were observed in the presence of K_2_CO_3_ and TlOAc. Finally, exclusive and high yielding access to the 6,6,5 architecture was obtained using a combination of TlOAc, dppf and pentamethylpiperidine as the base.

The development of a shortcut sequence implying a selective and rapid access to the α-ylidene γ-lactone intermediates bearing a bromine atom followed by an intramolecular Heck arylation is therefore highly desirable. In this communication, we describe a two-step strategy towards fused tricyclic architectures starting from α-bromomethylbutenolide. The key α-ylidene γ-lactone intermediate was obtained in the first step through a Barbier reaction which allowed installation of the *ortho*-bromoaromatics. These intermediates were subsequently subjected to intramolecular Heck reaction conditions. In our case, the presence of the OH group located at the benzylic site accounted for the selective preparation of the tricyclic 6,6,5 α-vinylidene γ-lactone or the 6,5,5 lactone motifs depending on the catalytic precursor/base combination (Figure 2c). The generation of tetracyclic analogues was then examined using the same strategy.

## 2. Results and Discussion

**Scheme 1:**
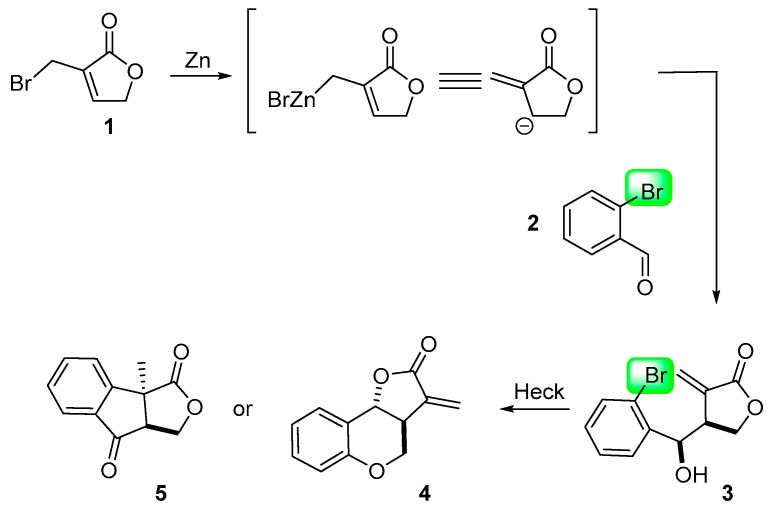
Route from α-bromomethylbutenolide to fused tricyclic lactones **4** and **5**.

We first examined the Barbier reaction between α-bromomethylbutenolide **1** and *ortho*-bromobenzaldehyde **2**. If such reactions are well described [3,4,5,6,7], the use of *ortho*-substituted benzaldehydes and further *ortho*-bromide derivatives remain scarcely reported [14]. In our case, **2** smoothly reacted with the starting butenolide at room temperature in THF for 16 h, in the presence of activated zinc powder (1.1 eq.) and saturated aq. NH_4_Cl as an additive. Under these conditions, homoallylic alcohol **3** was obtained at 65% with an 85:15 dr (Scheme 1).

The stereoselectivity of the major isomer is consistent with those described with other aromatic substrates [2], this was supported by our own NMR data (see ESI) and established by comparison with X-ray crystallographic analysis of the naphthalene analogue (vide infra). We next turned our attention to the intramolecular Heck cyclization under various conditions as exemplified in Table 1. 

**Scheme 2:**
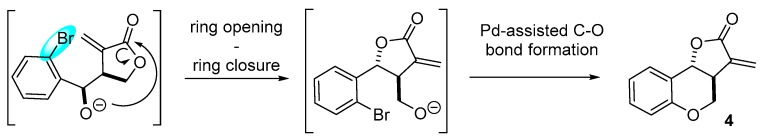
Plausible mechanism for the formation of lactone **4**.

Our first attempts were based on a Pd(II) catalytic system reported in the literature for an analogous transformation [3,4,5,6,7]. Pd(PPh_3_)_2_Cl_2_ (5 %) in combination with K_2_CO_3_ (2 eq.) was first used as the catalytic system in refluxing THF for two hours (entry 1). Although under these conditions, the reaction did not reach completion (see ratio of compounds 3/4/5 determined by ^1^H-NMR), we were able to isolate the unexpected tricyclic lactone **4** in 20% yield. The structure of **4** was unambiguously assigned by NMR experiments. Our strategy represents an alternative to the construction of fused tricyclic lactone architectures combining fused cyclopentenone and dihydrofuranone or γ-lactone-fused benzopyrans [8,9,15,16,17]. After 16 h, we noticed full conversion of the starting material and lactone **4** was isolated in a fair 60% yield together with some unidentified degradation material (entry 2). Under these conditions, no traces of the expected tricyclic 6,5,5 product was detected in the crude material. These first entries differ markedly from earlier observations within similar series [10,11,12]. Indeed, as described by Genet and Ikeda [10,11,12], the tricyclic products arising from the intramolecular Heck process is obtained either in the absence of a homoallylic hydroxyl group or in the presence of a Si-protected hydroxyl group (Figure 2a,b). In our case, the presence of an unprotected hydroxyl group allowed a different pathway to take place. As shown in Scheme 2, compound **4** and two new fused *O*-heterocycles might arise from a ring opening–ring closure sequence starting from the potassium alcoholate, through an intramolecular trans lactonization process, followed by an intramolecular Pd-assisted C-O bond formation [18]. Attempts to modify the reaction course by using silver salts [19] proved detrimental to the transformation only affording degradation material (entry 3). Changing from THF to MeCN as the solvent or Pd(PPh_3_)_2_Cl_2_/K_2_CO_3_ to Pd(PPh_3_)_2_Cl_2_/Cs_2_CO_3_ as the catalytic combination did not improve the isolated yield or led to degradation of the starting material respectively (entries 4 and 5). A control reaction run without a palladium source (entry 6) in order to isolate the intermediate from the ring opening–ring closure sequence failed, affording tangled mixtures of polycondensation products.

**Scheme 3:**
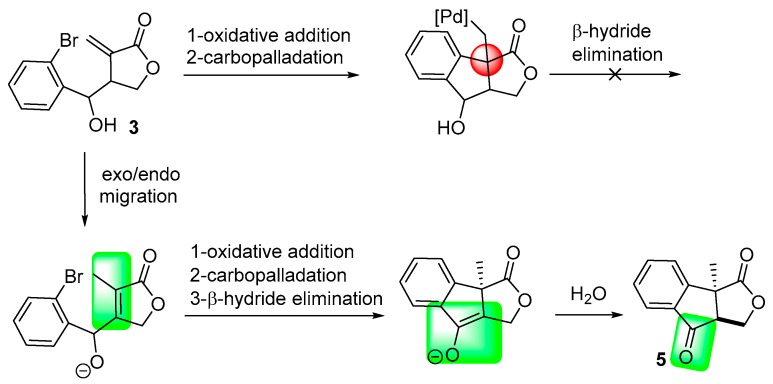
Plausible mechanism for the obtention of lactone **5**.

A combination of Pd(dppf)_2_Cl_2_/K_2_CO_3_ in MeCN or THF at reflux led to average yields of 30% and 45%, respectively, accompanied by degradation products (entries 7 and 8). Interestingly, the use of KOAc instead of K_2_CO_3_ allowed a complete switch of selectivity as tricyclic compound **4** was not detected (entry 9). Indeed, such conditions afforded a mixture of the starting material and a small amount of lactone **5**. Gratifyingly, we were able to cleanly isolate lactone **5** in a 40% yield by changing from THF to MeCN as the solvent (entry 10). It is worthy to note that the intramolecular Heck cyclization afforded the carbonyl compound **5** instead of the expected corresponding benzylic alcohol. Under our conditions, the formation of lactone **5** can be explained as shown in Scheme 3. The generation of a quaternary carbon center arising from oxidative addition and carbopalladation in a 5-*exo* process at precursor **3** precludes classical β-hydride elimination. The formation of lactone **5** could thus arise from an alternative pathway, involving an *exo*/*endo* migration of the olefin prior to Heck reaction. The α,β-unsaturated lactone thus generated would then successively undergo oxidative addition and carbopalladation in a 5-*endo* pattern followed by β-hydride elimination. The latter sequence would then generate an enolate and the corresponding ketone after aqueous workup.

**Scheme 4:**
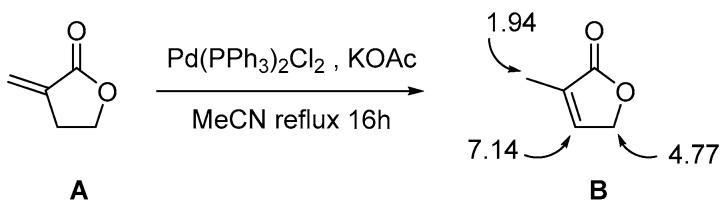
Exo/endo migration of the olefin from α-methylene-γ-butyrolactone (**A**) to the α,β-unsaturated lactone; (**B**) using Pd(PPh_3_)_2_Cl_2_/KOAc in refluxing MeCN.

This sequence requires an *exo*/*endo* migration of the olefin to take place prior the oxidative addition as the first key step leading to lactone **5**. The formation of allylic alcohols from homoallylic alcohols including homoallylic benzylic alcohols using Pd/C and Et_3_N has already been reported [20]. In addition, migration of the olefin from α-methylene-γ-butyrolactone to the corresponding α,β-unsaturated lactone has been obtained using RhCl_3_ in EtOH [21] and observed as a side product of cross metathesis reactions [22]. Unfortunately, we have not been able to demonstrate the olefin migration on the closely related dehalogenated analogue of compound **3** under our reaction conditions. However, in good agreement with the latter reports, we have been able to acquire evidence for the olefin migration of the olefin from α-methylene-γ-butyrolactone **A** to the corresponding α,β-unsaturated lactone **B** under our reaction conditions (Pd(PPh_3_)_2_Cl_2_/KOAc in refluxing MeCN) as shown in Scheme 4. The presence of characteristic signals of lactone **B** in the ^1^H-NMR of the crude material (see supplementary material) confirmed the olefin isomerization in full agreement with data reported by Jefford et al. [21].

The stereoselectivity was established by comparison with an X-ray crystallographic structure of the naphthalene analogue (vide infra). Again, the use of silver salts disappointingly afforded a sluggish reaction from which compound **5** could be isolated in 14% yield (entry 11). Finally, the use of a 1:1 mixture of K_2_CO_3_ and KOAc in refluxing THF afforded a partial conversion of the starting material **3** and formed lactone **4** as well as traces of lactone **5** in a 1/1.2/0.1 ratio (entry 12). Our results seem to indicate that both the nature of the base and the reaction conditions are essential to the selective transformation of benzylic alcohol **3**. Indeed, higher temperature in MeCN combined with the use of KOAc as the base affords the tricyclic 6,5,5 lactone **5**, whereas lower temperature in THF associated to K_2_CO_3_ affords the tricyclic 6,6,5 lactone **4**.

The same strategy was tested on *ortho*-bromobenzonitrile **6** and *ortho*-bromobenzaldimine **7**. Unfortunately, in both cases under similar Barbier conditions only the α-methylbutenolide arising from Zn-promoted reduction of the C-Br bond could be isolated (Table 2, entries 1 and 2). In contrast, moving from the phenyl to the commercially available naphthyl substrate **8** led to the formation of alcohol **12** in 60% yield with an 87:13 dr (entry 3). The rigid naphthalene fragment did not affect the dr observed for compound **3**. Moving towards the more flexible dihydronaphthalene platforms **9** and **10** [23] (entries 4 and 5) allowed preparation of the corresponding Barbier adducts **13** and **14** in higher yields ranging from 80 to 89% with similar drs of 87:13 and 94:6 regardless of the nature of the halide (Cl or Br) in the precursors.

**Figure 3:**
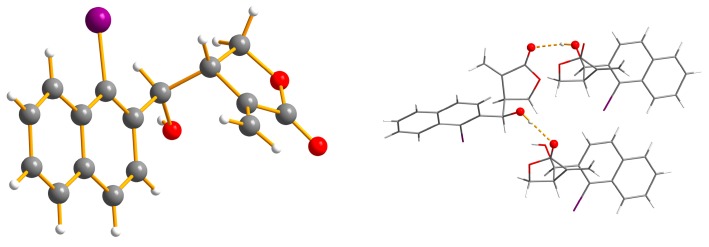
X-ray structure of **12** and diastereoselectivity [24].

X-ray crystallographic analysis undoubtedly assigned the stereoselectivity of the major isomer as shown in Figure 3. The presence of a sterically demanding naphthalene platform, as well as a bromine atom, did not affect the stereoselectivity observed for other aromatic substrates [3,4,5,6,7]. The combined presence of the lactone carbonyl and the benzyl alcohol induces the formation of hydrogen bonds associating three molecules in the solid state.

**Scheme 5:**
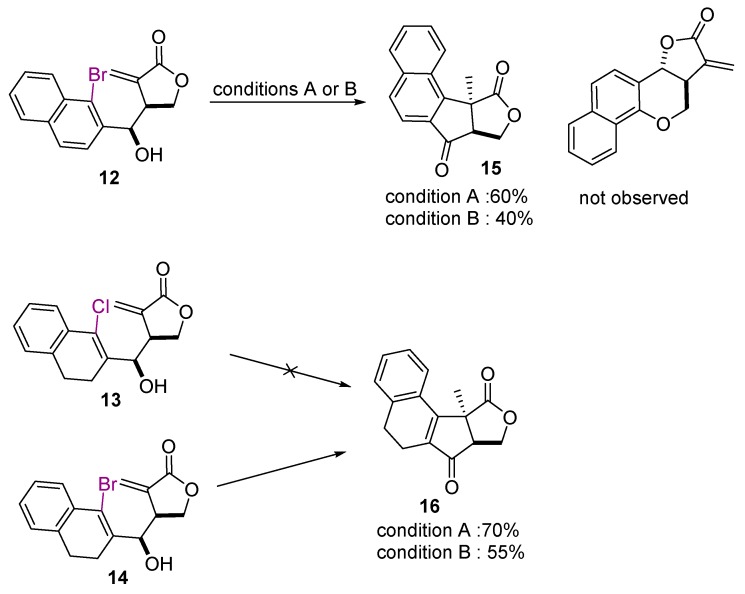
Synthesis of tetracyclic lactones **15** and **16**.

**Figure 4:**
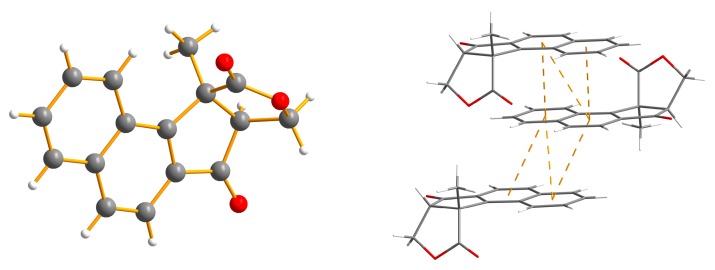
X-ray structure of tetracyclic lactone **16** [24].

Finally, (dihydro)naphthalene substrates **12**, **13**, and **14** were subjected to the aforementioned intramolecular Heck cyclization conditions. For the naphthyl substrate, conditions A (PdCl_2_(PPh_3_)_2,_ KOAc, MeCN at 90 °C for 18 h) and B (PdCl_2_(PPh_3_)_2,_ K_2_CO_3_, THF at 65 °C for 18 h) were tested. Interestingly, only the tetracyclic lactone **15** arising from a Heck cyclisation–oxidation sequence was isolated in 40% and 60% yields, respectively, under these reaction conditions. Further, no trace of the naphthyl analogue of compound 4 was observed even in the presence of K_2_CO_3_ as the base. At this stage, no satisfactory explanation for the unexpected selectivity observed towards the tetracyclic lactone **15** can be given. The reactivity of the dihydronaphthalene-based substrates **13** and **14** towards the Heck cyclization–oxidation sequence were next evaluated. Although no cyclization occurred using the less reactive chloride derivative **13**, the expected product **16** could be obtained in 55 to 70% yields from the bromide derivative **14**. Similarly, only one cyclization product was observed under both reaction conditions. Moving from the fully aromatic to the dihydro platform (compare **12** and **14**) had only a minor effect on the yield. Again, single crystal X-ray diffraction analysis confirmed the tetracyclic architecture of **16** and allowed assignment of the stereochemistry of the lactone–cyclopentanone junction (Scheme 5 and Figure 4).

As shown below, the crystalline lattice is formed by a pillared arrangement of tetracyclic units resulting from well-defined intermolecular π–π interactions (3.52 Å) between planar naphthalene fragments.

## 3. Materials and Methods

Unless otherwise noted, all starting materials were obtained from commercial suppliers and used without purification. Petroleum ether was distilled under Argon. NMR spectra were recorded on 300 MHz and 200 MHz Brucker spectrometers (Bruker BioSpin GmbH, Rheinstetten, Germany).

Chemical shifts were reported in ppm relative to the residual solvent peak (7.27 ppm for CHCl_3_ in the ^1^H-NMR and 77.0 ppm for CDCl_3_^13^C-NMR). High resolution mass spectroscopy data were recorded on an Autospec Ultima (Waters/Micromass) device (Waters, Gyancourt, France) with a resolution of 5000 RP at 5%. Thin-layer chromatography (TLC) was carried out on aluminium sheets precoated with silica gel 60 F254. Column chromatography separations were performed using silica gel (0.040–0.060 mm). Compound **1**, **7**, **9**, **10** and **11** were prepared according to the literature [25,26,27,28,29]. Compounds **6** and **8** are commercially available.

### 3.1. Methods

#### 3.1.1. Representative Procedure for the Barbier Allylation Reaction of 3-Bromomethyl-5*H*-furan-2-one

To a reaction vessel were added sequentially 3-bromomethyl-5*H*-furan-2-one **1** (400 mg, 2.26 mmol), aldehyde **2** (1.53 mmol, 0.68 eq.), THF (2 mL) saturated aqueous NH_4_Cl (1 mL) and activated zinc powder [30] (2.64 mmol, 1.17 eq.). The mixture was stirred vigorously at ambient temperature. After 16 h, the reaction was filtered through diatomite, extracted with diethyl ether (2 × 20 mL for each extraction), washed with brine (20 mL), and dried over anhydrous MgSO_4_. Evaporation in vacuo followed by flash column chromatography on silica gel (petroleum ether/ethyl acetate, 7:3) afforded homoallylic alcohols **3**, **12**, **13**, and **14**.

#### 3.1.2. Procedure A for Intramolecular Heck Reaction

A mixture of lactone **3** (100 mg, 0.35 mmol), PdCl_2_(PPh_3_)_2_ (12.5 mg, 0.017 mmol), and K_2_CO_3_ (98 mg, 0.71 mmol) in solvent (3 mL) was purged under argon atmosphere and stirred at 95 °C for 16 h. When the reaction was complete (as indicated by TLC), the mixture was diluted with water (10 mL) and extracted with CH_2_Cl_2_ (3 × 10 mL). The combined organic layers were dried over anhydrous MgSO_4_, concentrated under reduced pressure, and purified by flash column chromatography on silica gel eluted with CH_2_Cl_2_/petroleum ether (8:2) to give the expected product **4**.

#### 3.1.3. Procedure B for Intramolecular Heck Reaction

A mixture of lactone **3** (100 mg, 0.35 mmol), PdCl_2_(PPh_3_)_2_ (12.5 mg, 0.017 mmol), and KOAc (69.6 mg, 0.71 mmol) in CH_3_CN (3 mL). The mixture was purged under argon atmosphere and stirred at 95 °C for 16 h. When the reaction was complete (as indicated by TLC), the mixture was diluted with water (10 mL) and extracted with CH_2_Cl_2_ (3 × 10 mL). The combined organic layers were dried over anhydrous MgSO_4_ and concentrated under reduced pressure and purified by flash column chromatography on silica gel eluting with CH_2_Cl_2_/petroleum ether (8:2), to give the expected product

## 4. Conclusions

In summary, we have developed a three-step sequence involving a Barbier reaction followed by an intramolecular Heck arylation to prepare tri- and tetracyclic lactones starting from α-bromomethylbutenolide. The Zn-promoted allylation reaction proved efficient in the benzene, naphthalene, and dihydronaphthalene series giving access to various allylic/benzylic alcohols with high stereoselectivity. In the key Heck arylation step, the nature of the base proved crucial for obtaining polycyclic architectures. In the benzene series, switching from K_2_CO_3_ to KOAc selectively led to the expected tricyclic 6,5,5 pattern instead of the unexpected 6,6,5 pattern. Our strategy could be extended to tetracyclic analogues based on a naphthalene and a dihydronaphthalene platform.

## Figures and Tables

**Table 1:**
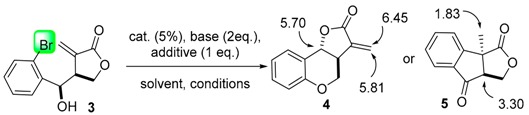
Selective access to lactones **4** and **5**.

Entry	Catalyst	Base	Additive	Solvent	Conditions	3/4/5 ^a^	Yield (%)
1	Pd(PPh_3_)_2_Cl_2_	K_2_CO_3_		THF	65 °C, 2 h	1/1/0	**4**(20)
2	Pd(PPh_3_)_2_Cl_2_	K_2_CO_3_		THF	65 °C, 16 h	0/1/0	**4**(60)
3	Pd(PPh_3_)_2_Cl_2_	K_2_CO_3_	Ag_2_CO_3_	THF	65 °C, 16 h	-	- ^c^
4	Pd(PPh_3_)_2_Cl_2_	K_2_CO_3_		MeCN	90 °C, 16 h	0/1/0	**4**(50)
5	Pd(PPh_3_)_2_Cl_2_	Cs_2_CO_3_		MeCN	90 °C, 2 h	-	- ^c^
6	-	K_2_CO_3_		THF	65 °C, 16 h	-	- ^d^
7	Pd(dppf)Cl_2_	K_2_CO_3_		MeCN	90 °C, 2 h	0.5/1/0	**4**(30)
8	Pd(dppf)Cl_2_	K_2_CO_3_		THF	65 °C, 16 h	0/1/0	**4**(45)
9	Pd(PPh_3_)_2_Cl_2_	KOAc		THF	65 °C, 16 h	1/0/0.1	nd
10	Pd(PPh_3_)_2_Cl_2_	KOAc		MeCN	90 °C, 16 h	0/0/1	**5**(40)
11	Pd(PPh_3_)_2_Cl_2_	KOAc	AgOAc	MeCN	90 °C, 16 h	0/0/1	**5**(14)
12	Pd(PPh_3_)_2_Cl_2_	KOAc, K_2_CO_3_^b^		THF	65 °C, 16 h	1/1.2/0.1	- ^a^

^a^ Ratio determined by NMR on crude products and compared integration of compounds **3**, **4**, and **5** characteristic signals (chemical shifts given in ppm); ^b^ Realised using 1 eq. of KOAc and 1 eq. of K_2_CO_3_; ^c^ Degradation; ^d^ Polycondensation products.

**Table 2:** Barbier reaction on substrates **6** to **10**.

Entry	Starting Halide	Compound	Condition	α-Methylidene Butyrolactone	Product	Yield (%)	Dr ^a^
1	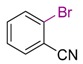	**6**	THF, 18 h	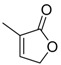	**11**	-	-
2	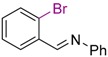	**7**	THF, 18 h		**11**	-	-
3	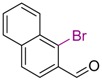	**8**	THF, 18 h	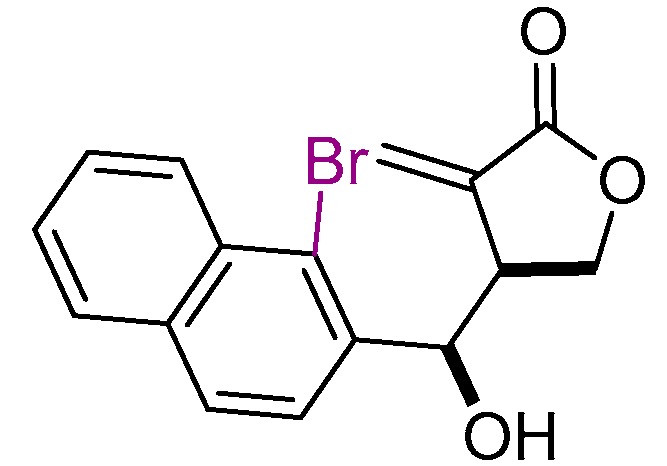	**12**	60	87/13
4	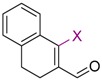	**9**, X = Cl	THF, 16 h	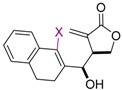	**13**, X = Cl	80	89/11
5		**10**, X = Br			**14**, X = Br	89	94/6

^a^ Determined on crude material NMR data.

## Supplementary Materials

Representative synthetic procedures, characterization data of new compounds, as well as NMR and X-ray data are available online, experimental procedures as well as analytical data for new compounds.
