# Supplementary material for: From α-Bromomethylbutenolide to Fused Tri(Tetra) Cyclic Dihydrofurandiones through Barbier Reaction–Heck Arylation Sequence
Source: Molecules. 2017 Dec 8;22(12):2171. doi: 10.3390/molecules22122171 (PMC6149967; doi:10.3390/molecules22122171)

Supporting Information

From α-bromomethylbutenolide to fused tri(four)cyclidihydro-furandiones through Barbier reaction-Heck arylation-oxidation sequence

Arbia Talbi,[a,b] Anne Gaucher,[b] Flavien Bourdreux,[b] Jérome Marrot,[b] Mohamed Lotfi Efrit,[a] Hédi M'Rabet,*[a] and Damien Prim*[b]

[a]Université de Tunis El Manar, Faculté des Sciences de Tunis, Laboratoire de Synthèse Organique Sélective et Hétérocyclique-Evaluation de l'Activité Biologique, 2092 Tunis, Tunisie
[b]Université de Versailles Saint Quentin-en-Yvelines, Institut Lavoisier de Versailles-UMR CNRS 8180, 45 avenue des Etats-Unis, 78035 Versailles, France

Corresponding author. Fax.: +33(0)1 39 25 44 52; damien.prim@.uvsq.fr

List of the Contents

1. General information
2. Representative procedure for allylation of aldehydes with 3-bromomethyl-5*H*-furan-2-one
3. Characterization data for the lactone 3
4. Synthetic procedure for Pd-catalyzed Heck reaction of bromoderivative 3
5. Characterization data for compounds 4 and 5
6. Synthetic procedure for Pd-catalyzed Heck reaction of bromoderivatives 12 -14
7. Characterization data for compounds 15 and 16
8. References
9. ^{1}H and ^{13}C-NMR Spectra
10. Noesy experiment results
11. control experiment : olefin migration
11. X-ray crystal structure for lactone 3 and 16

1. General information

All reagents and solvents were obtained from commercial sources and used without further purification. Reactions were routinely carried out under nitrogen and argon atmosphere with magnetic stirring. ^{1}H and ^{13}C-NMR spectra were recorded on a Bruker AMX 300 spectrometer working at 300 MHz, 75 MHz respectively for ^{1}H and ^{13}C with chloroform-d as solvent. Chemicals shifts were reported in δ, parts per million (ppm), relative to chloroform (δ = 7.28 ppm) as international standards unless otherwise stated for proton nuclear magnetic resonance (^{1}H-NMR). Chemical shifts for carbon nuclear magnetic resonance (^{13}C-NMR) were reported in δ, parts per million (ppm), relative to the center line of the chloroform triplet (δ = 77.07 ppm). Coupling constants, *J*, were reported in Hertz (Hz) and refer to apparent peak multiplicities and not true coupling constants. The abbreviations s, d, dd, t, q, br and m stand for resonance multiplicities singlet, doublet, doublet of doublet,

triplet, quartet, broad and multiplet, respectively. Allylation diastereoselectivity was determined by ^{1}H-NMR integrations of the methylene signals in the crude products. High Resolution Mass spectroscopy data were recorded on an Autospec Ultima (Waters/Micromass) device with a resolution of 5000 RP at 5%. Thin-layer chromatography (TLC) was carried out on aluminium sheets precoated with silica gel plates (Fluka Kiesel gel 60 F254, Merck) and visualized by a 254 nm UV lamp and potassium permanganate. Melting points (Mp) were determined on a System Kofler type WME apparatus.

2. Representative procedure for allylation of aldehydes with 3-bromomethyl-5*H*-furan-2-one

To a reaction vessel were added sequentially 3-bromomethyl-5*H*-furan-2-one **1** (400 mg, 2.26 mmol), aldehyde **2** (1.53 mmol, 0.68 equiv.), THF (2 mL) saturated aqueous NH_4Cl (1 mL) and activated zinc powder [30] (2.64 mmol, 1.17 equiv.). The mixture was stirred vigorously at ambient temperature. After 16h, the reaction was filtered through diatomite, extracted with diethyl ether (2 x 20 mL for each extraction), washed with brine (20 mL) and dried over anhydrous $MgSO_4$. Evaporation *in cacuo* followed by flash column chromatography on silica gel (petroleum ether/ethyl acetate, 7:3) afforded homoallylic alcohols **3**, **12**, **13** and **14**

3. Characterization data for the lactone 3, 12, 13, 14

4-((2-Bromophenyl)(hydroxyl)methyl)-3-methylenedihydrofuran-2(3*H*)-one 3. White solid; Mp: 111-115 °C; Yield: (281, 65%); dr: (85:5); ^{1}H-NMR (300 MHz, $CDCl_3$): δ = 7.55 (d, *J* = 7.5 Hz, 1H), 7.46 (d, *J* = 7.8 Hz, 1H), 7.34 (t, *J* = 7.5 Hz, 1H), 7.18 (t, *J* = 7.5 Hz, 1H), 6.23 (d, *J* = 2.1 Hz, 1H), 5.22 (d, *J* = 2.4 Hz, 1H), 5.21 (d, *J* = 3.90 Hz, 1H), 4.34-4.32 (m, 2H), 3.52-3.48 (m, 1H), 2.98 (s (*br*.), 1H) ppm; Discernable data for minor diastereoisomer: δ = 6.28 (d, *J* = 2.4 Hz, 1H), 5.48 (d, *J* = 2.1 Hz, 1H), 5.25 (d, *J* = 4.8 Hz, 1H), 4.51 (dd, *J* = 9.3 Hz, *J* = 4.20 Hz, 1H), 4.19-4.13 (m, 1H) ppm; ^{13}C-NMR (75 MHz, $CDCl_3$): δ = 171.0 (*C*=O), 139.5 (*aromatic* =*C*), 133.7 (H_2C=*C*), 132.9 (*aromatic* =*C*H), 129.7 (*aromatic* =*C*H), 128.8 (*aromatic* =*C*H), 127.6 (*aromatic* =*C*H), 125.4 (*aromatic* =*C*-Br), 122.0 (H_2*C*=C), 74.0 (*C*H-OH), 68.5 (O*C*H_2), 43.6 (*C*H) ppm; HRMS (ESI) calcd. for $C_{12}H_{12}O_3Br$ $[M+H]^+$ 282.9970, found 282.9979.

4-((1-Bromonaphthalen-2-yl)(hydroxyl)methyl)-3-methylenedihydrofuran-2(3*H*)-one 12. Light yellow solid; Mp: 140-142 °C; Yield: (305 mg, 60%); dr: (87:13); ^{1}H-NMR (300 MHz, $CDCl_3$): δ = 8.35 (d, *J* = 8.4 Hz, 1H), 7.86 (d, *J* = 8.4 Hz, 2H), 7.63-7.59 (m, 3H), 6.34 (d, *J* = 2.1 Hz, 1H), 5.56 (d, *J* = 6.6 Hz, 1H), 5.48 (d, *J* = 1.5 Hz, 1H), 4.31-4.29 (m, 2H), 3.63-3.56 (m, 1H), 2.71 (s (*br*.), 1H) ppm; Discernable data for minor diastereoisomer: δ = 6.28 (d, *J* = 2.4 Hz, 1H), 5.60 (d, *J* = 5.4 Hz, 1H), 5.38 (d, *J* = 2.4 Hz, 1H), 4.65 (dd, *J* = 9.6 Hz, *J* = 4.2 Hz, 1H), 4.31-4.25 (m, 1H) ppm; ^{13}C-NMR (75 MHz, $CDCl_3$): δ = 170.9 (*C*=O), 137.6 (H_2C=*C*), 134.4 (*aromatic* =*C*), 134.3 (*aromatic* =*C*), 132.0 (*aromatic* =*C*), 128.2 (*aromatic* =*C*H), 128.1 (*aromatic* =*C*H), 127.9 (*aromatic* =*C*H), 127.5 (*aromatic* =*C*H), 127.2 (*aromatic* =*C*H), 125.7 (*aromatic* =*C*H), 125.1 (*aromatic* =*C*-Br), 122.5 (H_2*C*=C), 74.8 (*C*H-OH), 68.2 (O*C*H_2), 44.2 (*C*H) ppm; HRMS (ESI) calcd. for $C_{16}H_{14}O_3Br$ $[M+H]^+$ 333.0126, found 333.0138.

4-((1-Chloro-3,4-dihydronaphthalen-2-yl)(hydroxyl)methyl)-3-methylenedihydrofuran-2(3*H*)-one 13. Colorless oil; Yield: (357 mg, 80%); dr: (89:11); ^{1}H-NMR (300 MHz, $CDCl_3$): δ = 7.67-7.64 (m, 1H), 7.30-7.27 (m, 2H), 7.26-7.19 (m, 1H), 6.41 (d, *J* = 2.1 Hz, 1H), 6.10 (d, *J*= 1.5 Hz, 1H), 5.09 (d, *J* = 9.6 Hz, 1H), 4.25 (t, *J* =9.6 Hz, 1H), 4.10 (dd, *J* = 9.6 Hz, *J* = 4.5 Hz, 1H), 3.35-3.32 (m, 1H), 2.89-2.81 (m, 2H), 2.72-2.62 (m, 1H), 2.44 (s (*br*.), 1H), 2.39-2.28 (m, 1H) ppm; Discernable data for minor diastereoisomer: δ = 6.30 (d, *J* = 2.4 Hz, 1H), 5.57 (d, *J* = 2.1 Hz, 1H), 4.57 (dd, *J* = 9.3 Hz, *J* = 4.20 Hz, 1H), 4.47 (t, *J* = 9.3 Hz, 1H) ppm; ^{13}C-NMR (75 MHz, $CDCl_3$): δ = 170.7 (*C*=O), 136.3 (*aromatic* =*C*), 135.4 (H_2C=*C*), 134.7 (*aromatic* =*C*), 132.2 (Cl-C=*C*), 128.7 (*aromatic* =*C*H), 128.5 (*aromatic* =*C*H), 127.2 (*aromatic* =*C*H), 126.9 (=*C*-Cl), 125.6 (*aromatic* =*C*H), 125.1 (H_2C=C), 72.1 (*C*H-OH), 67.0 (O*C*H_2), 42.2 (*C*H), 27.7 (*C*H_2), 23.4 (*C*H_2) ppm; HRMS (ESI) calcd. for $C_{16}H_{16}O_3Cl$ $[M+H]^+$ 291.0782, found 291.0776.

4-((1-Bromo-3,4-dihydronaphthalen-2-yl)(hydroxyl)methyl)-3-methylenedihydrofuran-2(3*H*)-one 14. Grayish solid; Mp: 135-137 °C; Yield: (457 mg, 89%); dr: (94:6); ^{1}H-NMR (300 MHz, $CDCl_3$): δ = 7.67-7.66 (m, 1H), 7.30-7.23 (m, 3H), 7.17-7.15 (m, 1H), 6.43 (d, *J* = 2.1 Hz, 1H), 6.10 (d, *J* = 1.5 Hz, 1H), 5.09 (d, *J* = 9.6 Hz, 1H), 4.26 (t, *J* = 9.3 Hz, 1H), 4.13 (dd, *J* = 9.6 Hz, *J* = 4.6 Hz, 1H), 3.37-3.34 (m, 1H), 2.92-2.81 (m, 2H), 2.72-2.62 (m, 1H), 2.37-2.26 (m, 1H) ppm; ^{13}C-NMR (75 MHz, $CDCl_3$): δ = 170.6 (*C*=O), 137.9 (H_2C=*C*), 136.4 (Br-C=*C*), 135.3 (*aromatic* =*C*), 133.1 (*aromatic* =*C*), 128.8 (*aromatic* =*C*H), 127.7 (*aromatic* =*C*H), 127.1 (*aromatic* =*C*H), 127.0 (*aromatic* =*C*H), 125.6 (H_2*C*=C), 121.6 (=*C*-Br), 75.1 (*C*H-OH), 66.9 (O*C*H_2), 42.2 (*C*H), 27.8 (*C*H_2), 24.5 (*C*H_2) ppm; HRMS (ESI) calcd. for $C_{16}H_{16}O_3Br$ $[M+H]^+$ 335.0277, found 335.0271.

4. General procedure for Pd-catalyzed Heck reaction of bromoderivative 3

Procedure A

A mixture of lactone **3** (100 mg, 0.35 mmol), $PdCl_2(PPh_3)_2$ (12.5 mg, 0.017 mmol), and K_2CO_3 (98 mg, 0.71 mmol) in solvent (3mL) was purged under argon atmosphere and stirred at 95 °C for 16 h. After completion of the reaction (as monitored by TLC), the mixture was diluted with water (10 mL) and extracted with CH_2Cl_2 (3 x 10 mL). The combined organic layers were dried over anhydrous $MgSO_4$, concentrated under reduced pressure and purified by flash column chromatography on silica gel eluting with CH_2Cl_2/petroleum ether, (8:2) to give the expected product **4**.

3-Methylene-3a,9b-dihydro-4*H*-furo[3,2-c]chromen-2(3*H*)-one 4. Yellow oil; Yield: (29 mg, 40%); ^{1}H-NMR (300 MHz, $CDCl_3$): δ = 7.60 (d, *J* = 7.8 Hz, 1H), 7.39-7.20 (m, 3H), 6.47 (d, *J* = 2.1 Hz, 1H), 5.81 (d, *J* = 1.8 Hz, 1H), 5.71 (d, *J* = 3.3 Hz, 1H), 4.10 (dd, *J* = 12.0 Hz, *J* = 6.0 Hz, 1H), 3.97 (dd, *J* = 9.0 Hz, *J* = 6.0 Hz, 1H), 3.18-3.11 (m, 1H) ppm; ^{13}C-NMR (75 MHz, $CDCl_3$): δ = 170.0 (*C*=O), 138.8 (*aromatic =C*), 135.3 (H_2C=*C*), 133.2 (*aromatic =C*H), 129.9 (*aromatic =C*H), 128.0 (*aromatic =C*), 126.8 (*aromatic =C*H), 124.7 (H_2*C*=C), 121.1 (*aromatic* =CH), 79.4 (O*C*H), 64.0 (O*C*H_2), 49.6 (=C-*C*H) ppm; HRMS (ESI) calcd. for $C_{12}H_{11}O_3$ $[M+H]^+$ 203.0708, found 203.0706.

(10a)-Methyl-7a,10a-dihydro-7*H*-benzo[6,7]indeno[1,2-c]furan-7,10(8*H*)-dione 15. Yellow solid; Mp: 206-207 °C; Yield: (46 mg, 60%); ^{1}H-NMR (300 MHz, $CDCl_3$): δ = 8.95 (d, *J* = 9.9 Hz, 1H), 7.99-7.94 (m, 2H), 7.78-7.72 (m, 3H), 4.77 (t, *J* = 9.3 Hz, 1H), 4.45 (dd, *J* = 9.9 Hz, *J* = 5.10 Hz, 1H), 3.47 (dd, *J* = 10.5 Hz, *J* = 5.1 Hz, 1H), 2.08 (s, 3H) ppm; ^{13}C-NMR (75 MHz, $CDCl_3$): δ = 202.9 (*C*=O), 176.5 (*C*O_2), 153.9 (*aromatic =C*), 138.2 (*aromatic =C*), 133.0 (*aromatic =C*), 131.4 (*aromatic =C*), 129.7 (*aromatic =C*H), 129.6 (*aromatic =C*H), 129.3 (*aromatic =C*H), 127.8 (*aromatic =C*H), 127.6 (*aromatic =C*H), 119.2 (*aromatic =C*H) , 66.4 (O*C*H_2), 56.3 (*C*H), 52.0 (*C*CH_3), 23.5 (*C*H_3) ppm; HRMS (ESI) calcd. for $C_{16}H_{11}O_3$ $[M-H]^-$ 251.0708, found 251.0710.

(10a)-Methyl-6,7a,8,10a-tetrahydro-7*H*-benzo[6,7]indeno[1,2-c]furan-7,10(5*H*)-dione 16. Yellow solid; Mp: 122-124 °C; Yield: (46 mg, 60%); ^{1}H-NMR (300 MHz, $CDCl_3$): δ = 8.42 (d, *J* = 9.3 Hz, 1H), 7.40-7.30 (m, 3H), 4.64 (t, *J* = 9.9 Hz, 1H), 4.50 (dd, *J* = 9.9 Hz, *J* = 3.9 Hz, 1H), 3.19 (dd, *J* = 9.6 Hz, *J* = 3.6 Hz, 1H), 2.97-2.91 (m, 2H), 2.73-2.64 (m, 1H), 2.49-2.37 (m, 1H), 1.87 (s, 3H) ppm; ^{13}C-NMR (75 MHz, $CDCl_3$): δ = 203.5 (*C*=O), 176.3 (*C*O_2), 165.2 (=*C*-CO), 139.9 (*aromatic =C*), 138.0 (*aromatic =C*), 131.3 (=*C*), 129.3 (*aromatic =C*H), 128.7 (*aromatic =C*H), 128.5 (*aromatic =C*H), 127.1 (*aromatic =C*H), 66.4 (O*C*H_2), 55.4 (*C*CH_3), 52.0 (*C*H), 28.1 (*C*H_2), 22.0 (*C*H_2), 18.4 (*C*H_3) ppm; HRMS (ESI) calcd. for $C_{16}H_{15}O_3$ $[M+H]^+$ 255.1011, found 255.1016.

Procedure B

A mixture of lactone **3** (100 mg, 0.35 mmol), $PdCl_2(PPh_3)_2$ (12.5 mg, 0.017 mmol) and KOAc (69.6 mg, 0.71 mmol) in CH_3CN (3mL). The mixture was purged under argon atmosphere and stirred at 95 °C for 16 h. After completion of the reaction (as monitored by TLC), the mixture was diluted with water (10 mL) and extracted with CH_2Cl_2 (3 x 10 mL). The combined organic layers were dried over anhydrous $MgSO_4$ and concentrated under reduced pressure and purified by flash column chromatography on silica gel eluting with CH_2Cl_2/petroleum ether, (8:2) to give the expected product **5**.

(3a)-Methyl-3a,8a-dihydro-1*H*-indeno[1,2-c]furan-3,8-dione 5. Yellow solid; Mp: 122-124 °C; Yield: (29 mg, 40%). ^{1}H-NMR (300 MHz, $CDCl_3$): δ = 7.83-7.74 (m, 3H), 7.55 (d, *J* = 7.2 Hz, 1H), 4.66 (t, *J* = 9.9 Hz, 1H), 4.51 (dd, *J* = 9.9 Hz, *J* = 7.2 Hz, 1H), 3.30 (dd, *J* = 9.0 Hz, *J* = 3.3 Hz, 1H), 1.83 (s, 3H) ppm; ^{13}C-NMR (75 MHz, $CDCl_3$): δ = 203.4 (*C*=O), 177.2 (*C*O_2), 154.2 (*aromatic =C*), 136.5 (*aromatic =C*), 134.4 (*aromatic =C*H), 129.6 (*aromatic =C*H), 125.1 (*aromatic =C*H), 124.1(*aromatic =C*H), 66.4 (O*C*H_2), 54.7 (*C*CH_3), 29.6 (*C*H), 22.2 (*C*H_3) ppm; HRMS (ESI) calcd. for $C_{12}H_{11}O_3$ $[M+H]^+$ 203.0708, found 203.0706.

9. ^{1}H and ^{13}C-NMR Spectra

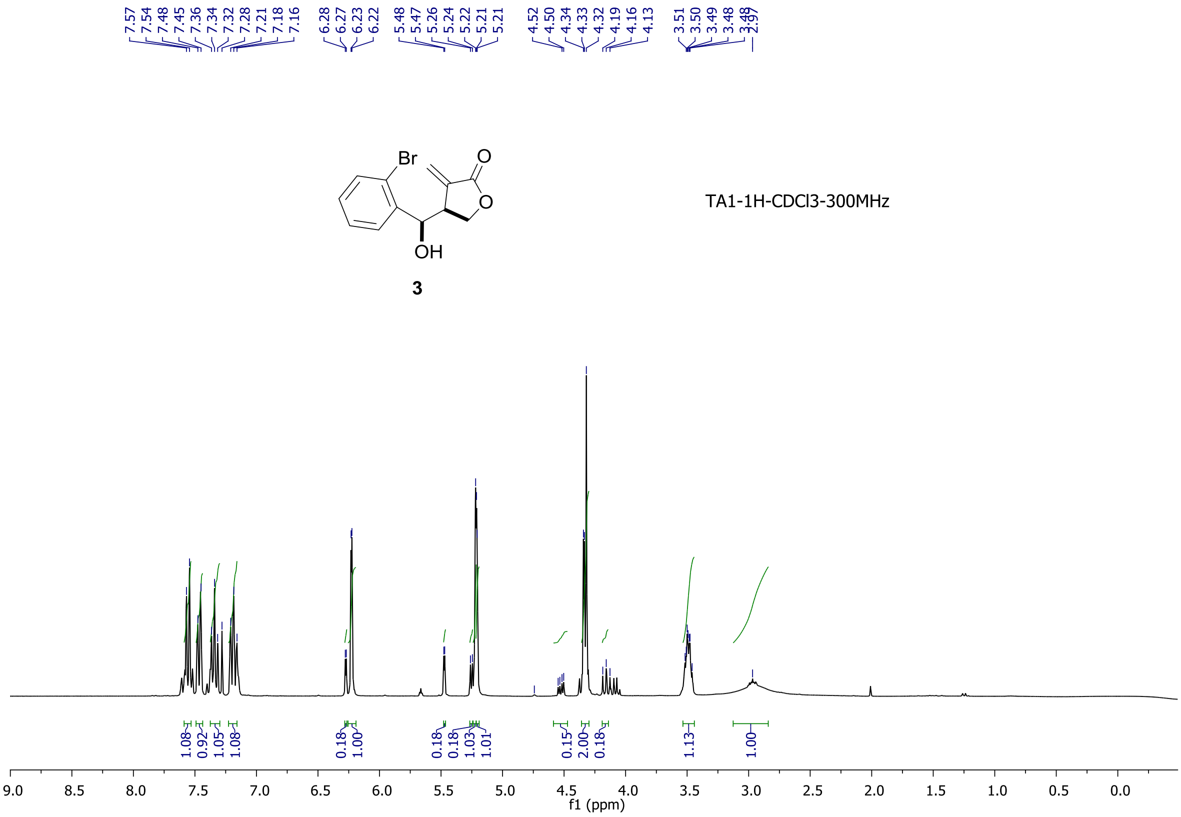

^{1}H-NMR Spectrum of Compound **3**

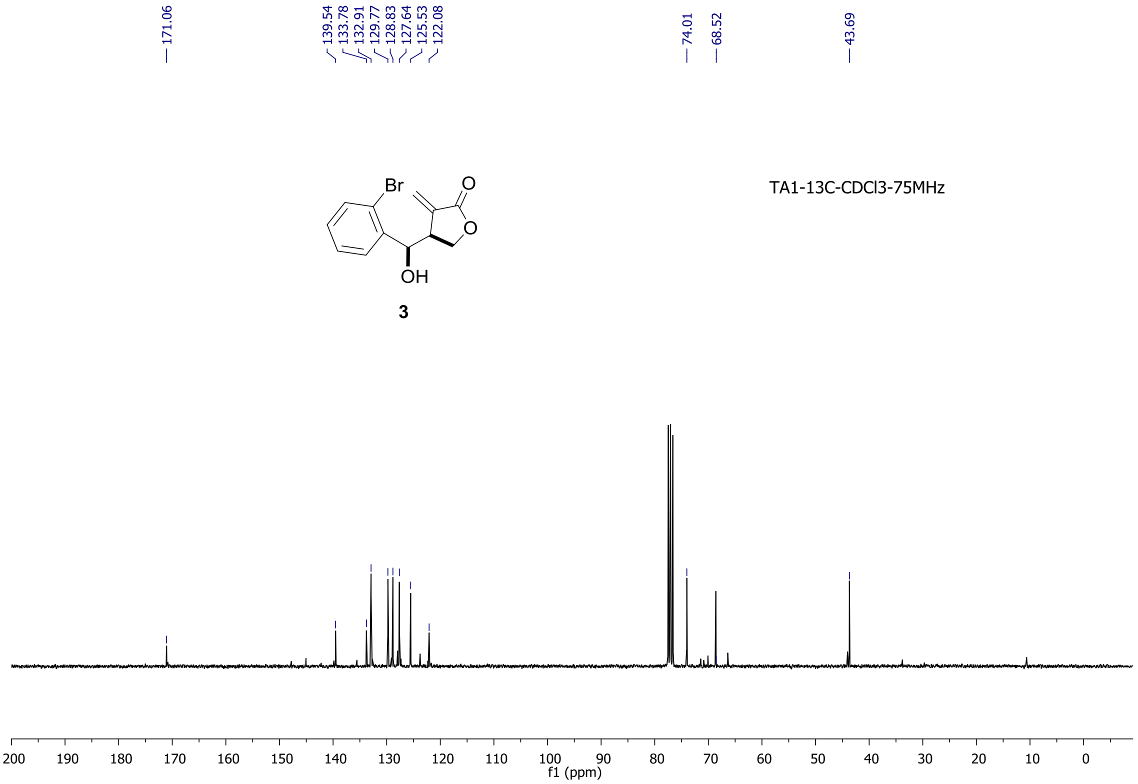

^{13}C-NMR Spectrum of Compound **3**

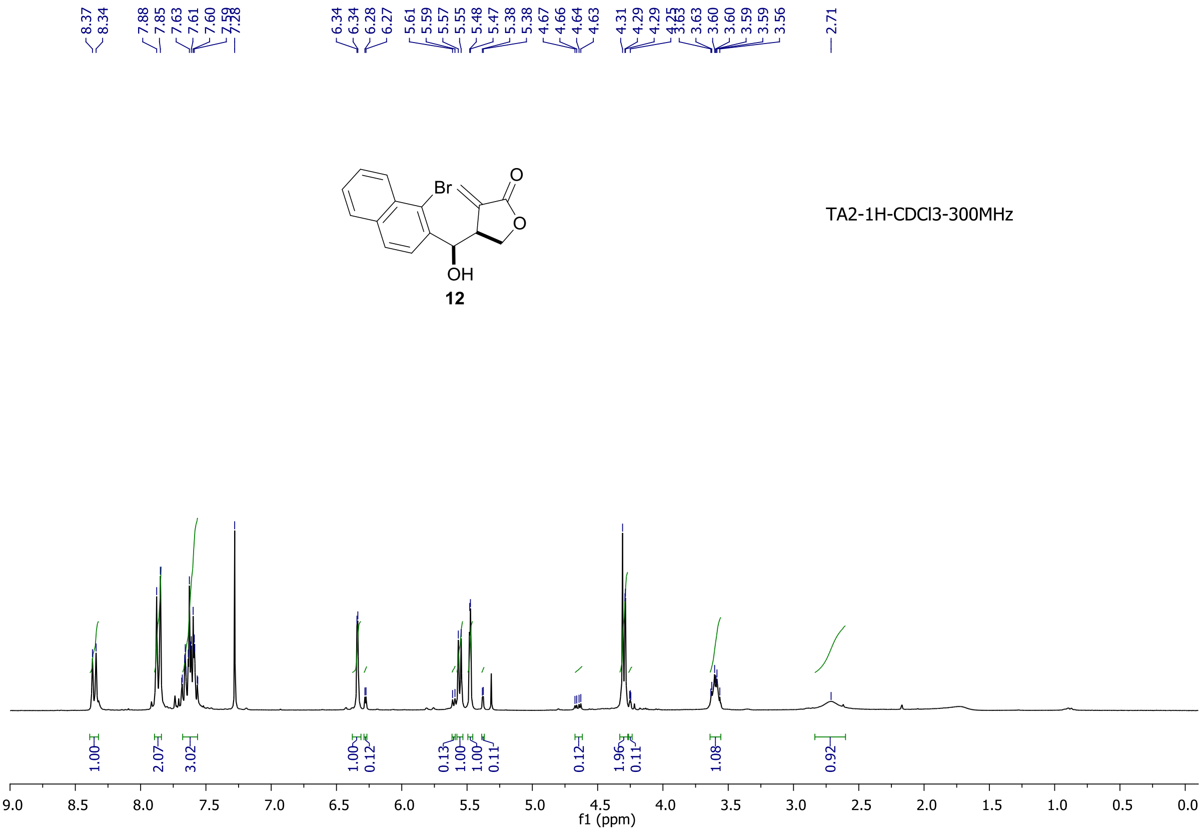

^{1}H-NMR Spectrum of Compound **12**

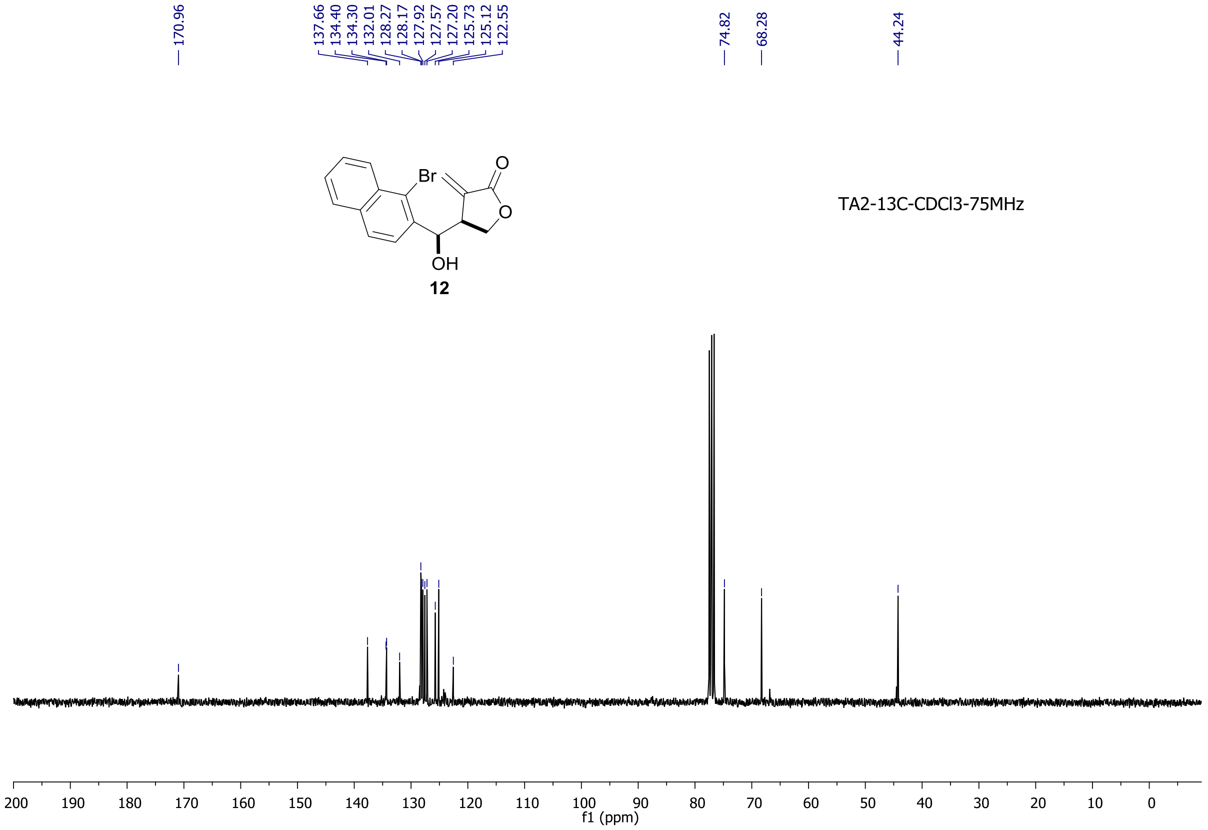

^{13}C-NMR Spectrum of Compound **12**

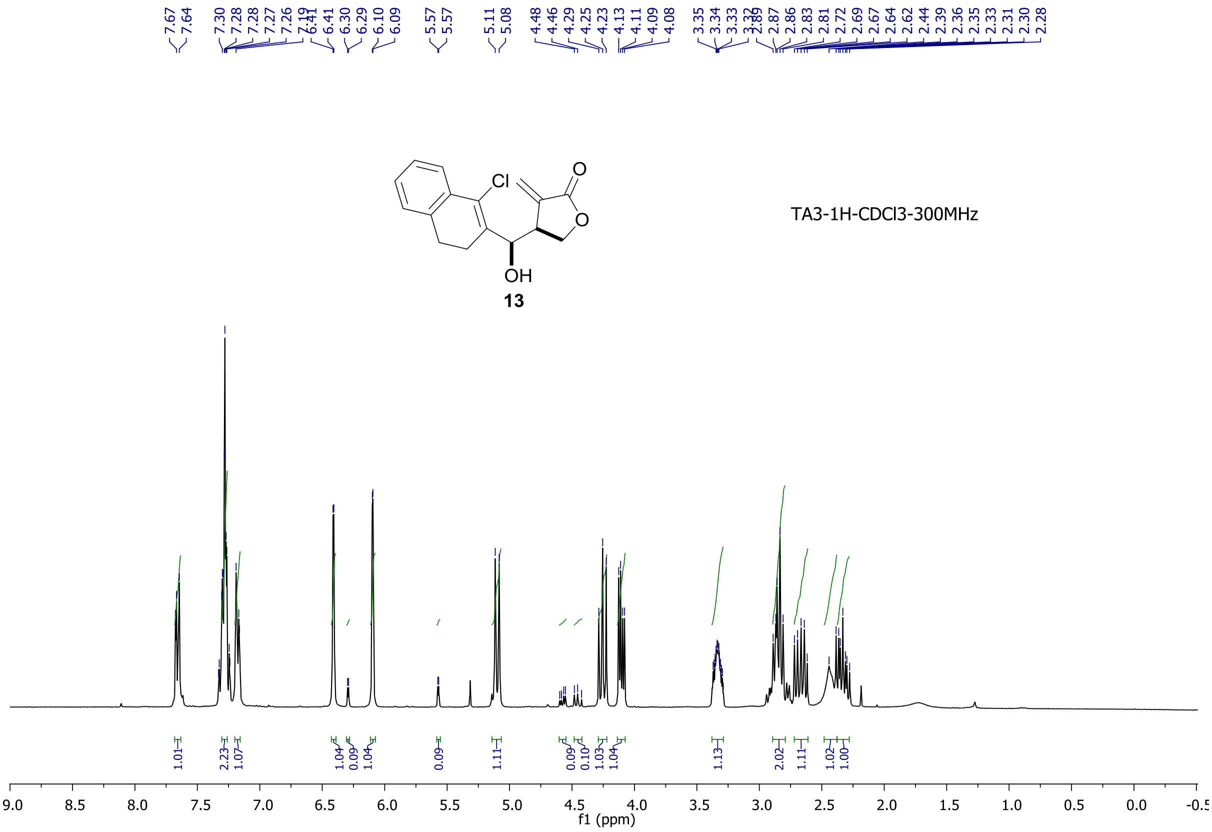

^{1}H-NMR Spectrum of Compound **13**

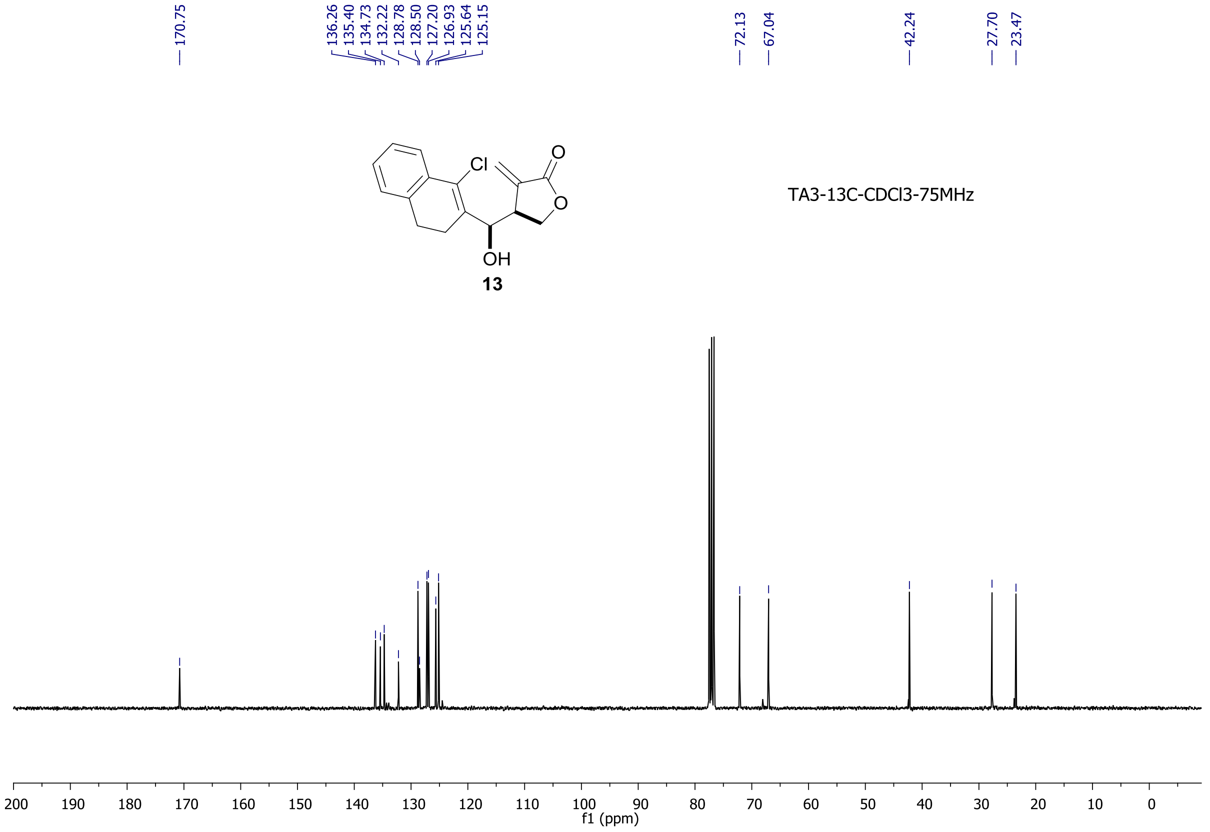

^{13}C-NMR Spectrum of Compound **13**

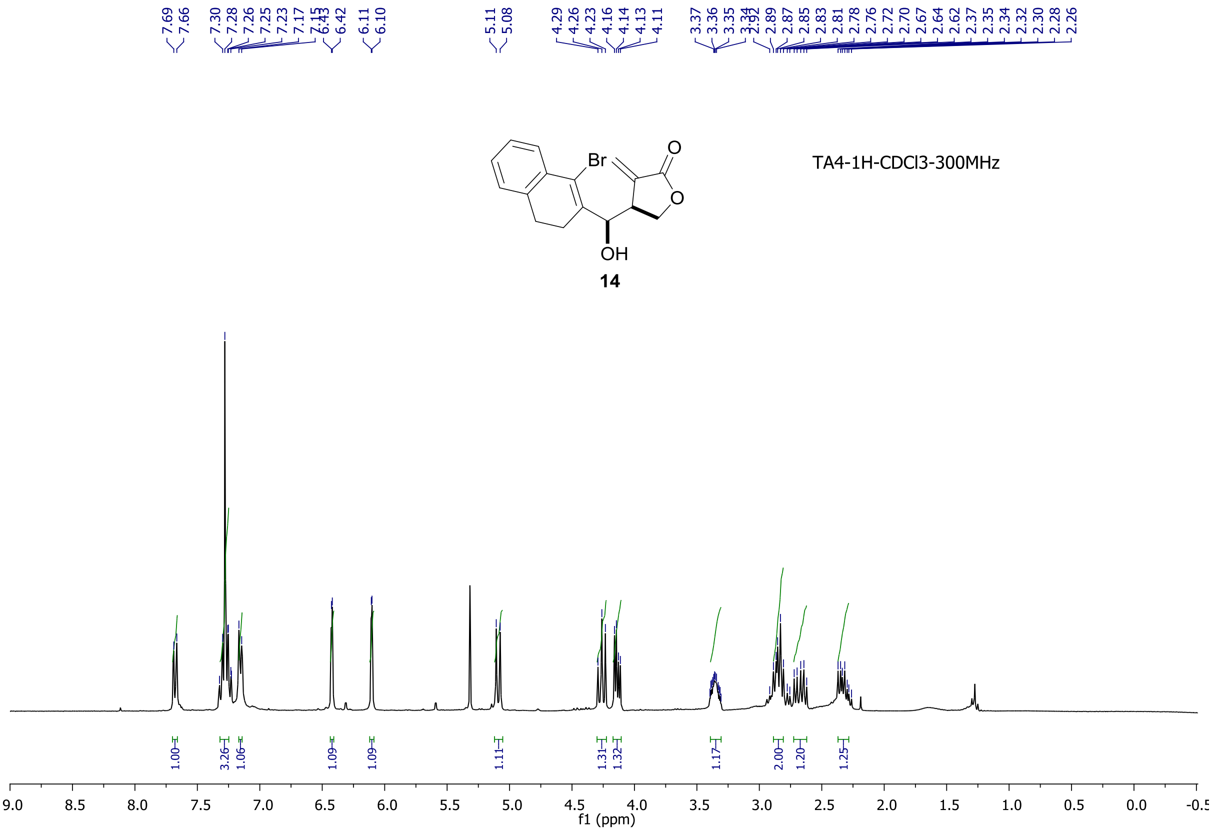

^{1}H-NMR Spectrum of Compound **14**

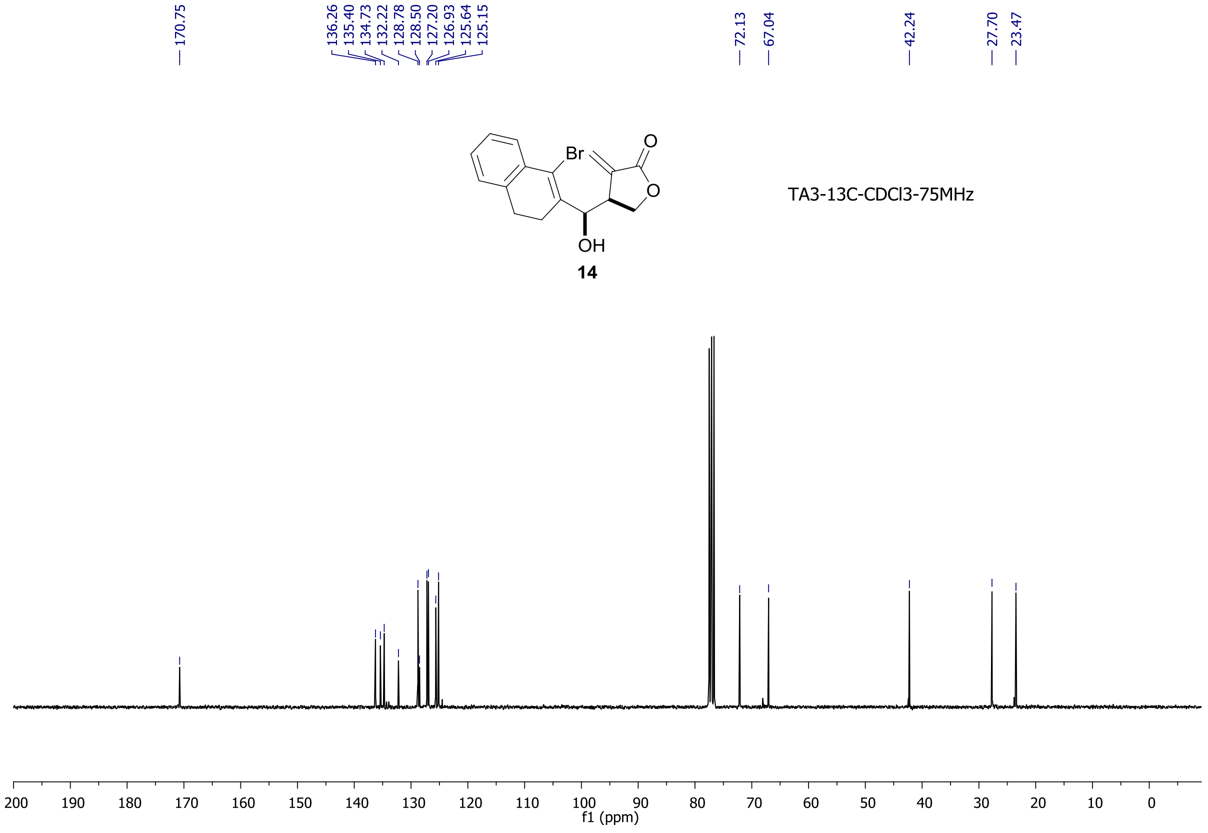

^{13}C-NMR Spectrum of Compound **14**

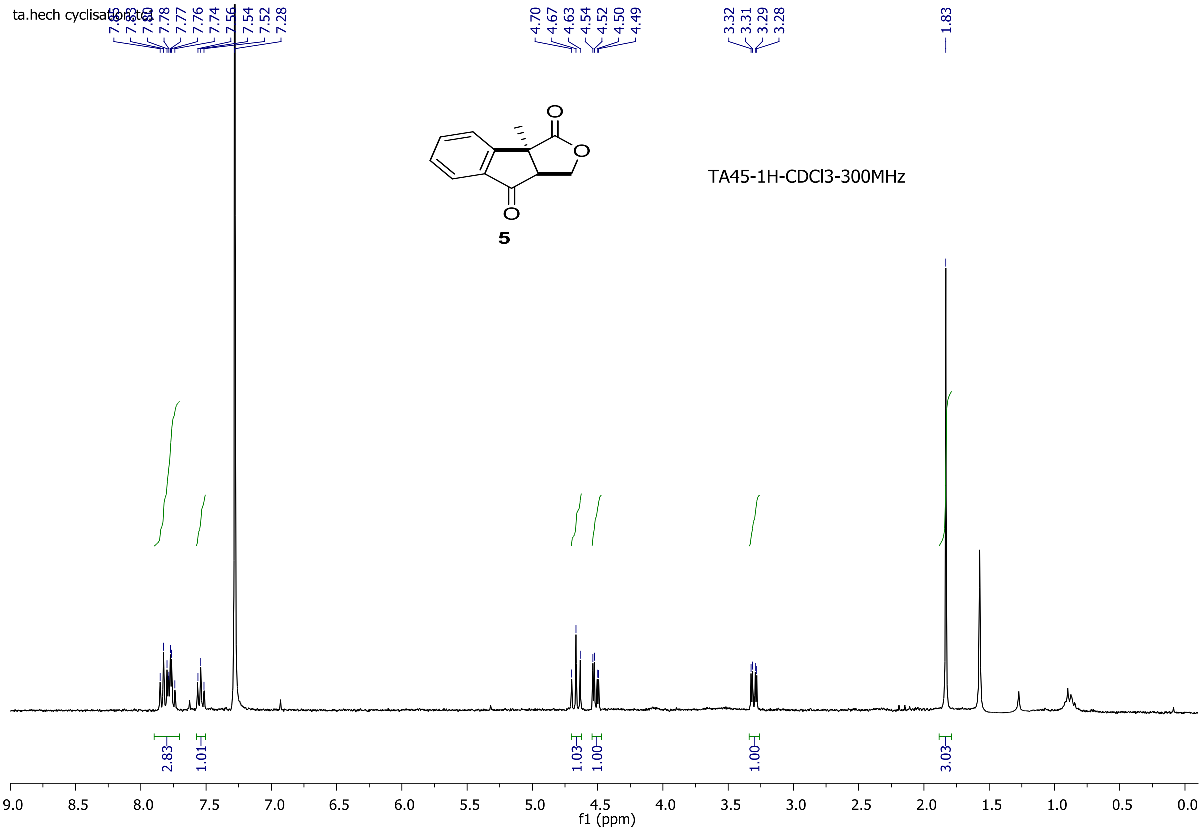

^{1}H-NMR Spectrum of Compound **5**

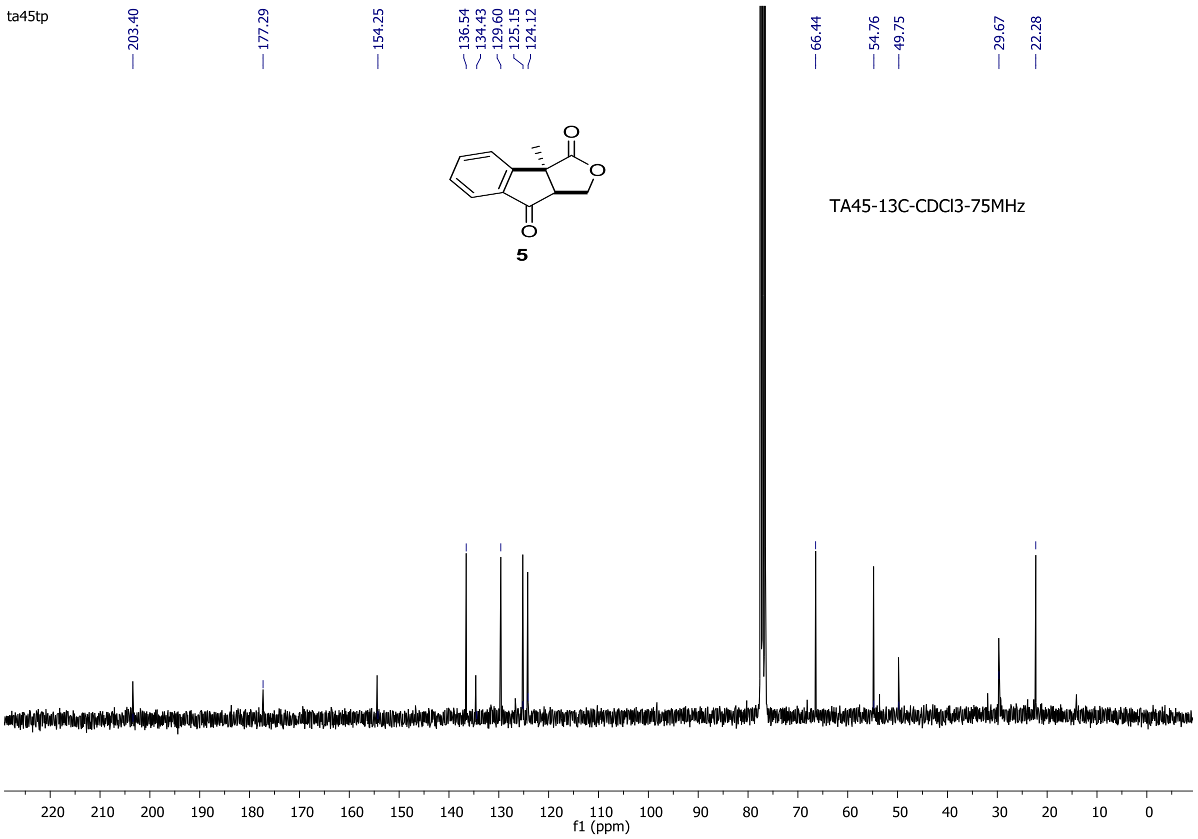

^{13}C-NMR Spectrum of Compound **5**

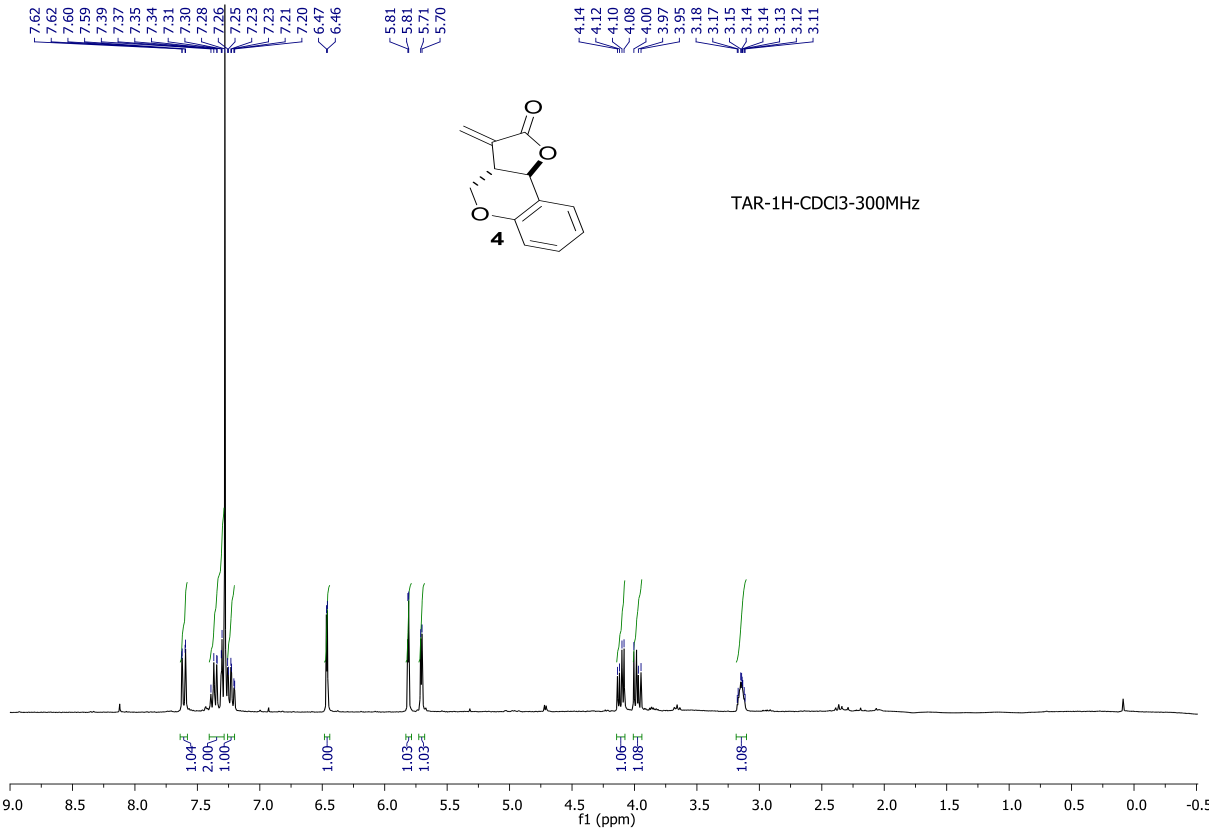

^{1}H-NMR Spectrum of Compound **4**

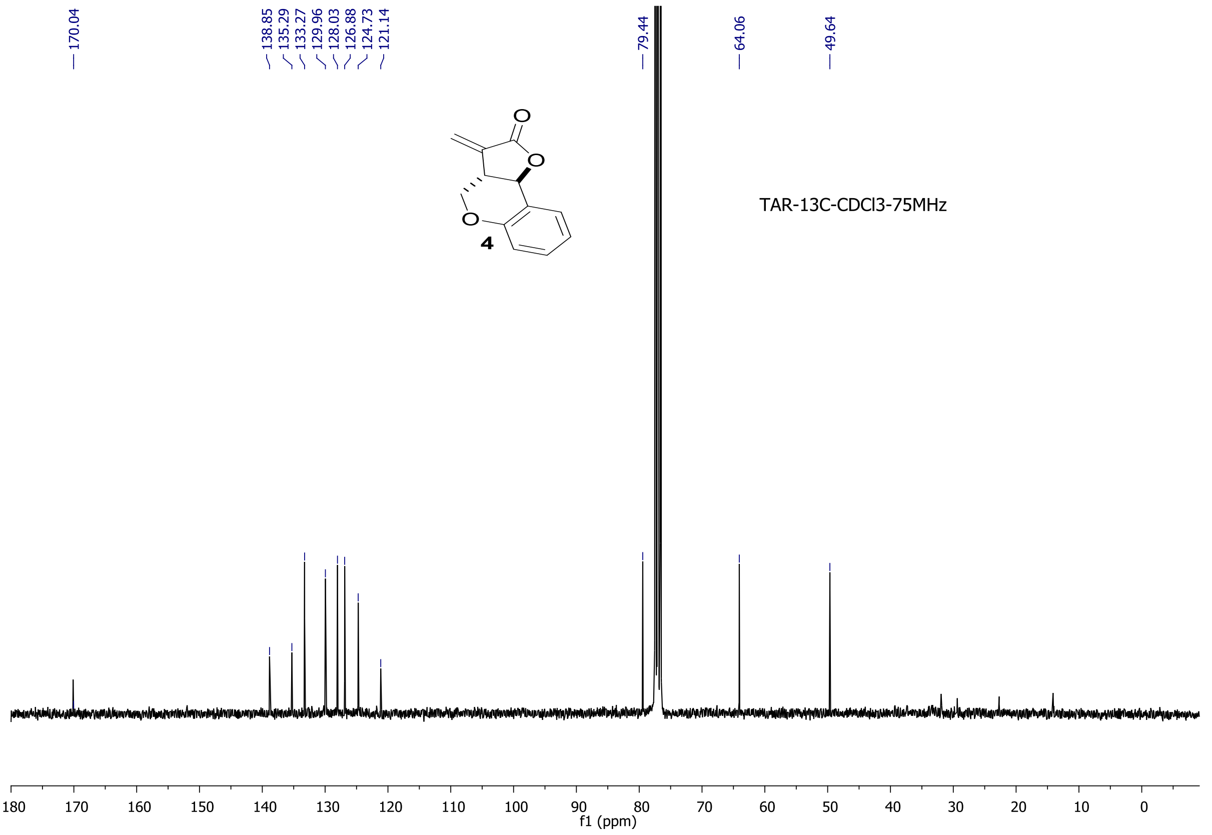

^{13}C-NMR Spectrum of Compound **4**

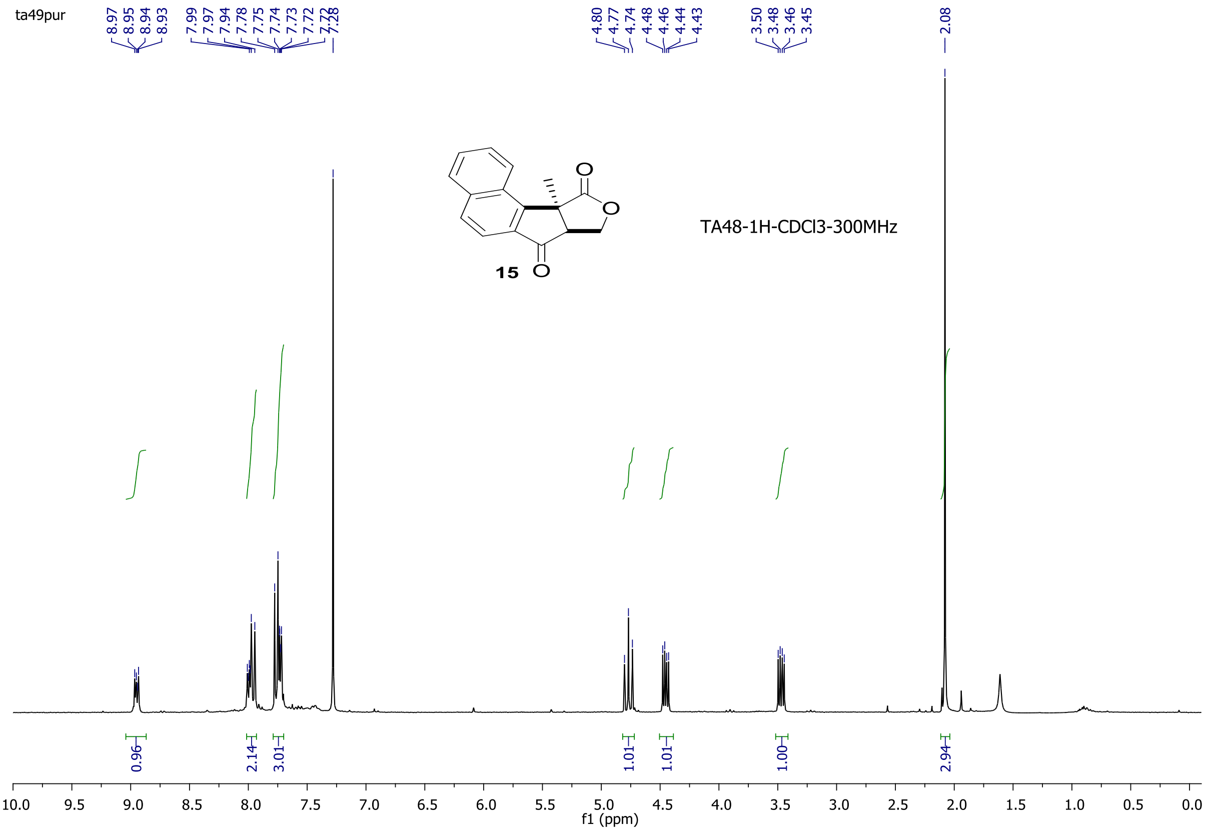

^{1}H-NMR Spectrum of Compound **15**

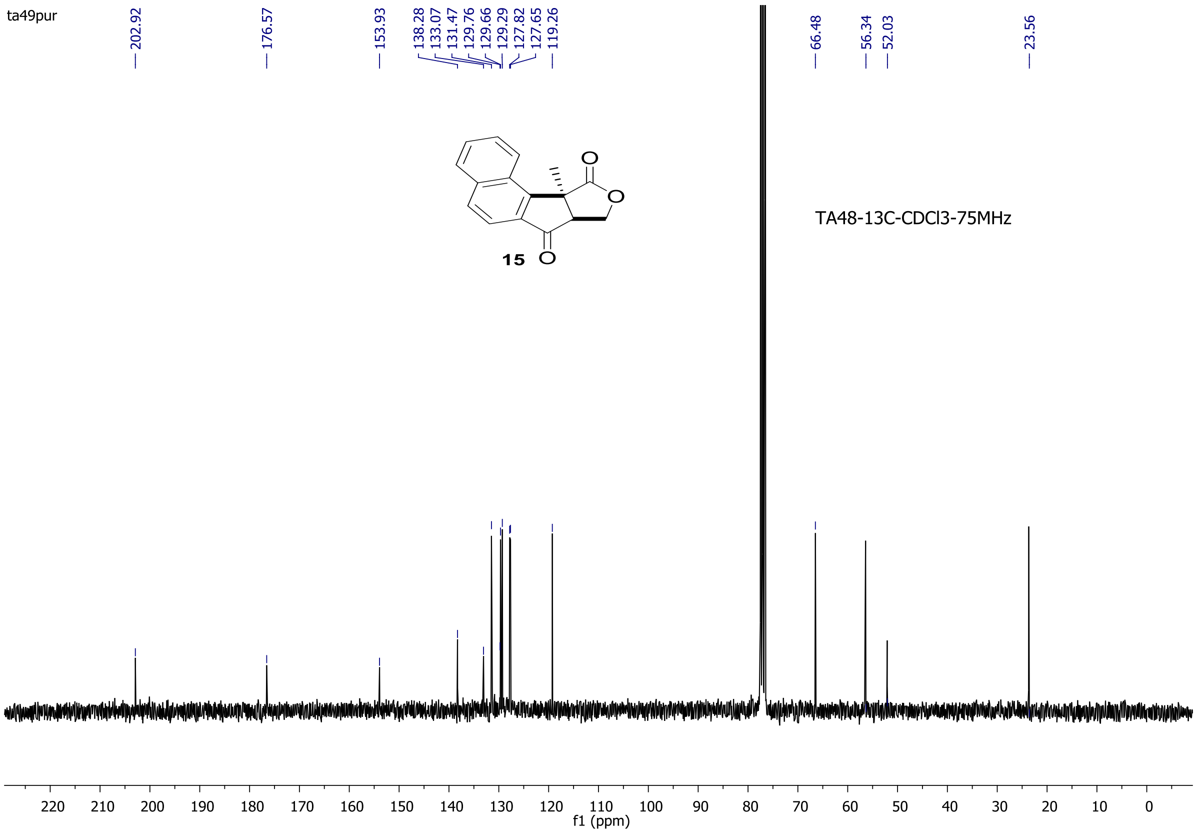

^{13}C-NMR Spectrum of Compound **15**

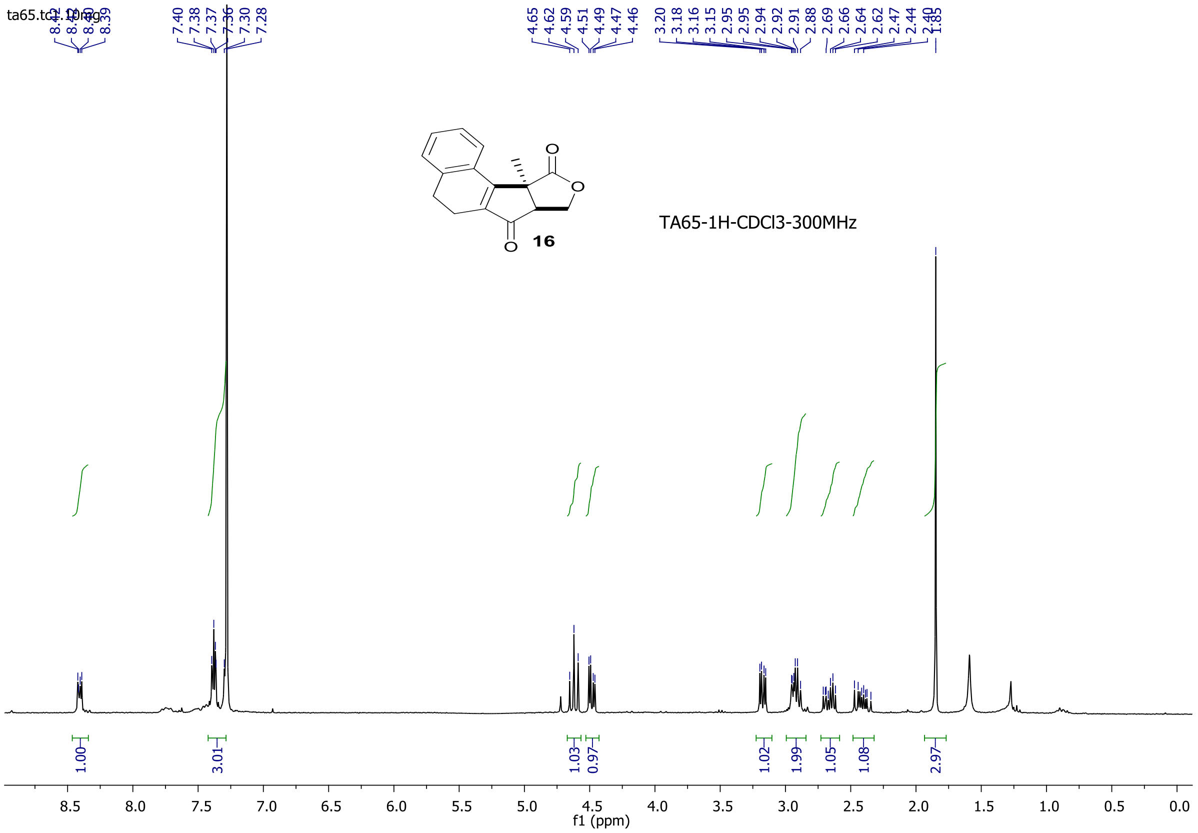

^{1}H-NMR Spectrum of Compound **16**

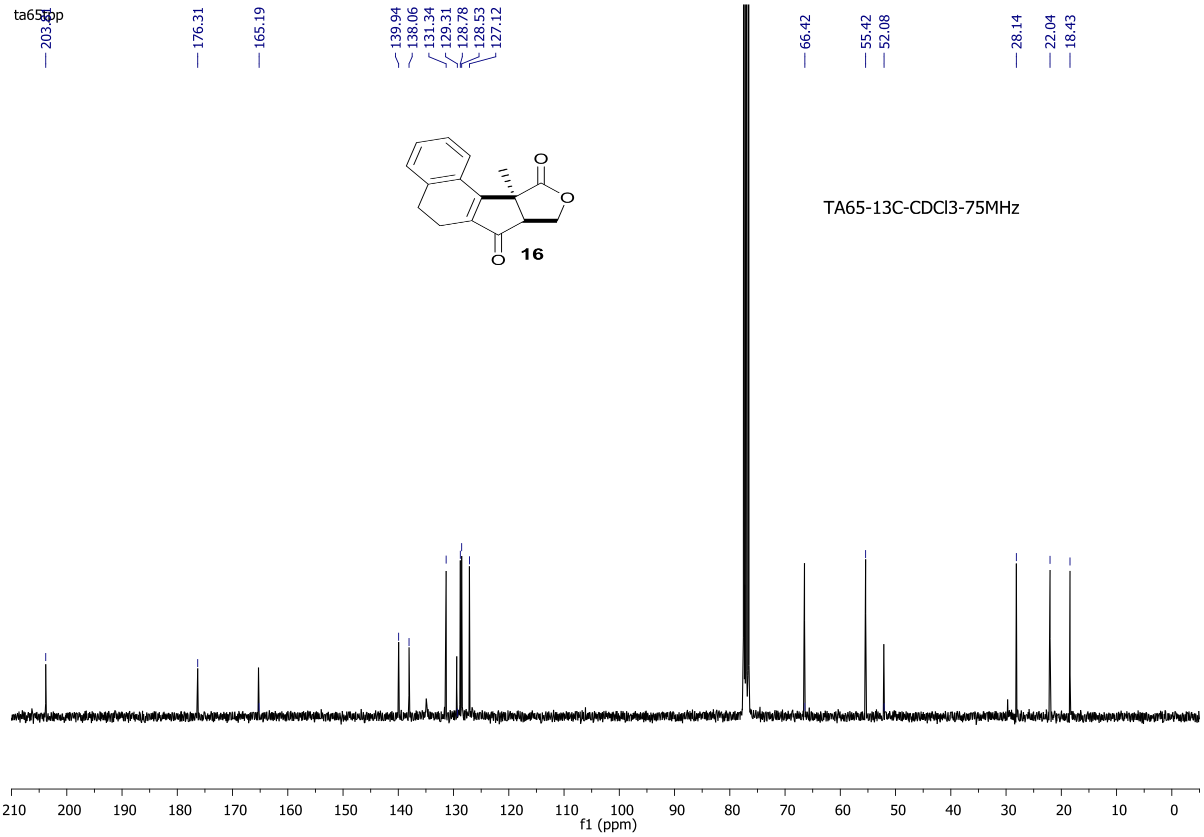

^{13}C-NMR Spectrum of Compound **16**

10. Noesy experiments: compound 4

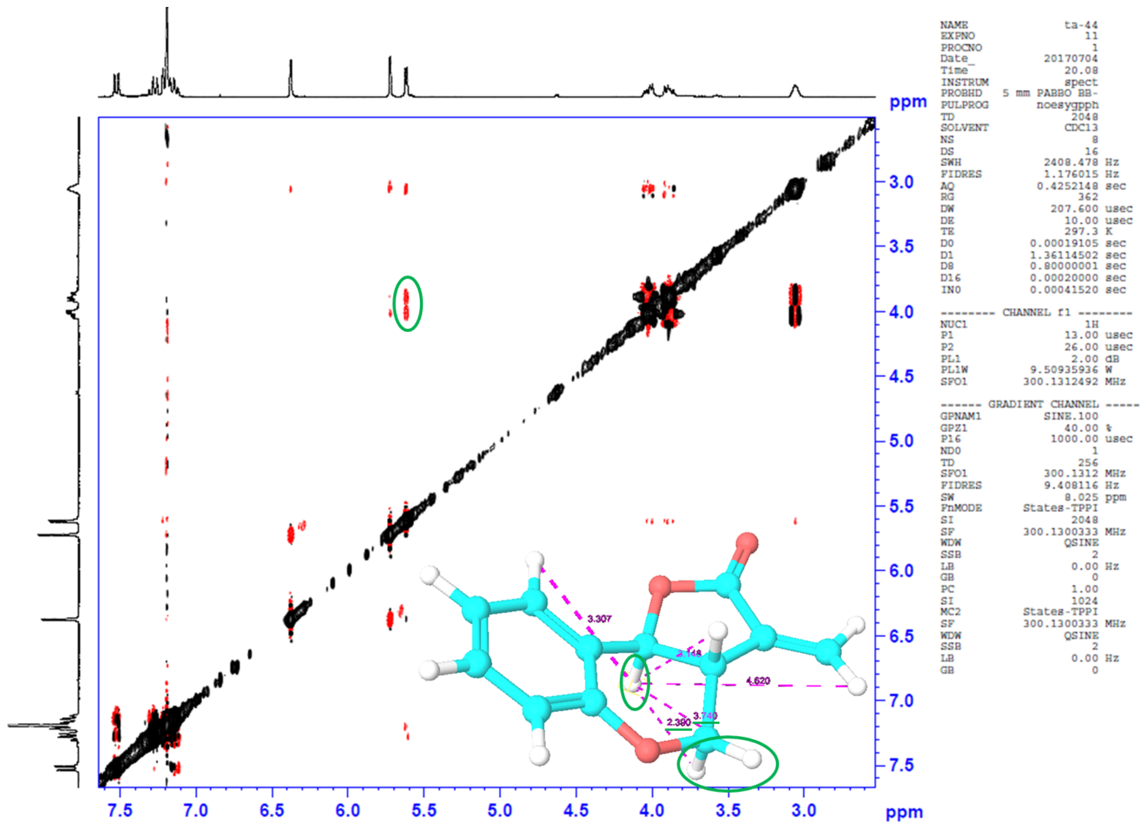

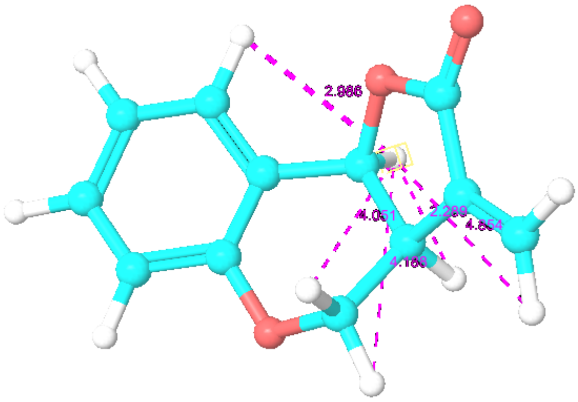

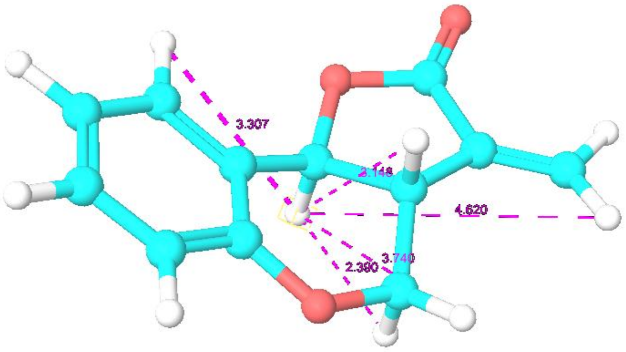

11. Control experiment : olefin migration

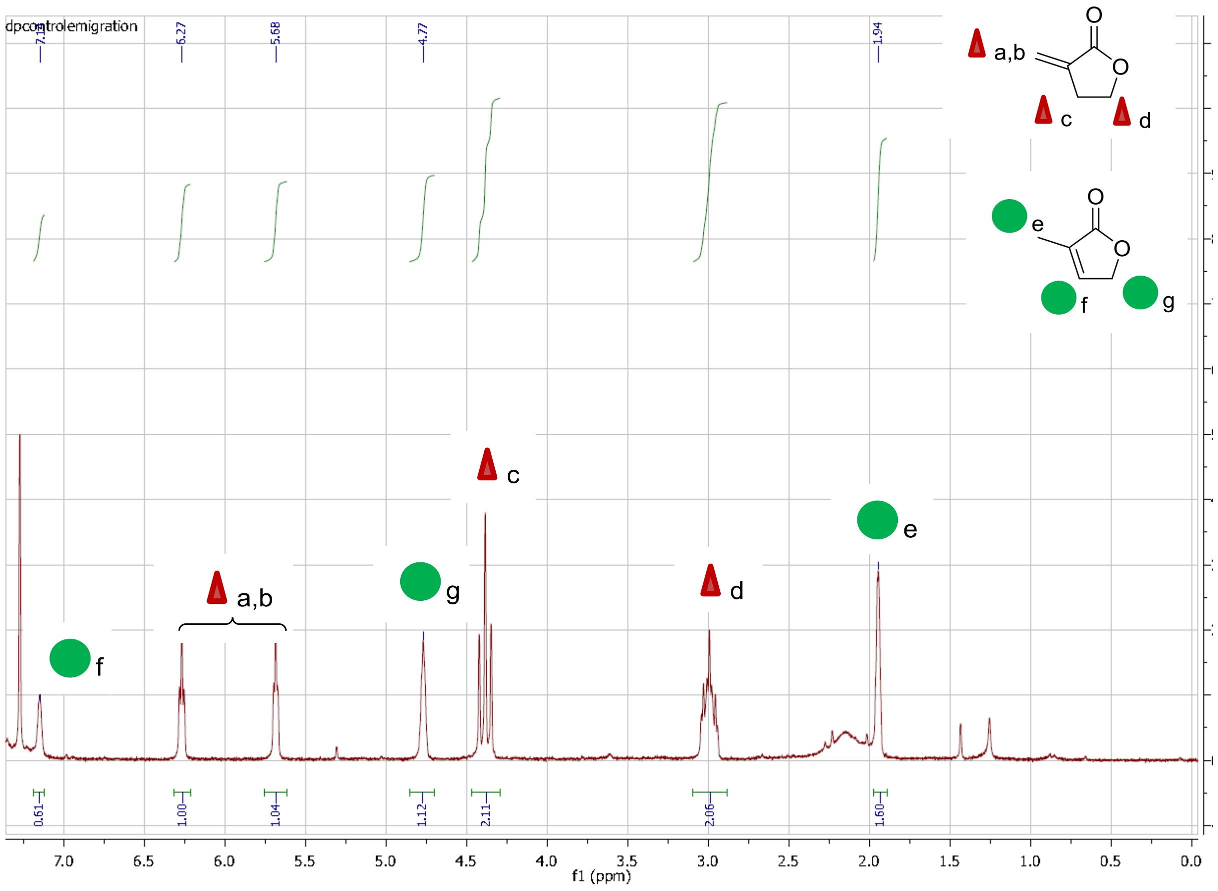

Chemical shifts in agreement with Jefford, C.W.; Rossier, J.-C.; Boukouvalas, J.; Sledeski, A.W.; Huang, P.-Z. A concise synthesis of siphonodictidine. *J. Nat. Prod.* **2004,** *67*, 1383–1386. DOI: 10.1021/np0400860.

12. X-ray crystal structure for lactone 12 and 16

Structure of 12 CCDC 1565162

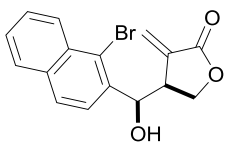

13

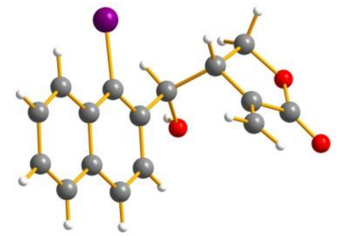

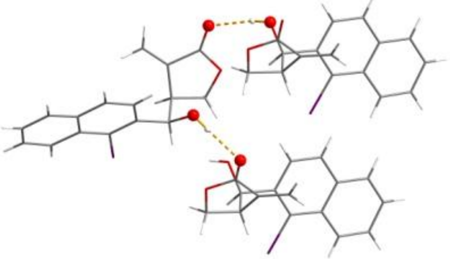

Structure of 16 CCDC 1565163

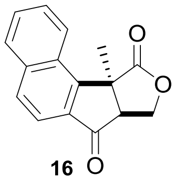

16

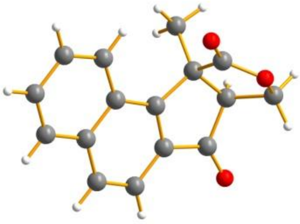

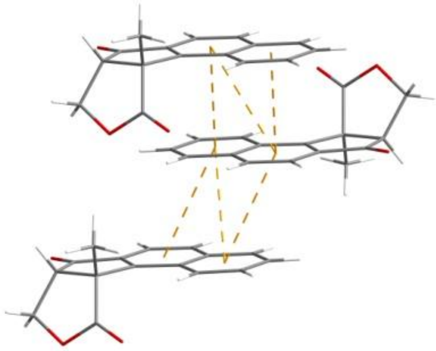

Supplement: Supplementary file 1 [file molecules-22-02171-s001.pdf]
